# Loss of transient receptor potential channel 5 causes obesity and postpartum depression

**DOI:** 10.1016/j.cell.2024.06.001

**Published:** 2024-07-02

**Authors:** Yongxiang Li, Tessa M. Cacciottolo, Na Yin, Yang He, Hesong Liu, Hailan Liu, Yuxue Yang, Elana Henning, Julia M. Keogh, Katherine Lawler, Edson Mendes de Oliveira, Eugene J. Gardner, Katherine A. Kentistou, Panayiotis Laouris, Rebecca Bounds, Ken K. Ong, John R.B. Perry, Inês Barroso, Longlong Tu, Jonathan C. Bean, Meng Yu, Kristine M. Conde, Mengjie Wang, Olivia Ginnard, Xing Fang, Lydia Tong, Junying Han, Tia Darwich, Kevin W. Williams, Yongjie Yang, Chunmei Wang, Shelagh Joss, Helen V. Firth, Yong Xu, I. Sadaf Farooqi

**Affiliations:** 1 USDA/ARS Children’s Nutrition Research Center, Department of Pediatrics, Baylor College of Medicine, Houston, TX, USA; 2 University of Cambridge Metabolic Research Laboratories, Institute of Metabolic Science and NIHR Cambridge Biomedical Research Centre, Cambridge, UK; 3 Jan and Dan Duncan Neurological Research Institute, Department of Pediatrics, Baylor College of Medicine, One Baylor Plaza, Houston, TX 77030, USA; 4 Taizhou People’s Hospital, Medical School of Yangzhou University, Taizhou, Jiangsu, China; 5 MRC Epidemiology Unit, Institute of Metabolic Science and NIHR Cambridge Biomedical Research Centre, Cambridge, UK; 6 Exeter Centre of Excellence for Diabetes Research (EXCEED), University of Exeter Medical School, Exeter, UK; 7 Center for Hypothalamic Research, Department of Internal Medicine, University of Texas Southwestern Medical Center at Dallas, Dallas, TX 75390-9077, USA; 8 West of Scotland Regional Genetics Service, Queen Elizabeth University Hospital, Glasgow, UK; 9 Department of Clinical Genetics, Cambridge University Hospitals NHS Foundation Trust & Wellcome Sanger Institute, Cambridge, UK; 10 Department of Molecular and Cellular Biology, Baylor College of Medicine, Houston, TX, USA; 11 Department of Medicine, Baylor College of Medicine, Houston, TX, USA; 12 These authors contributed equally; 13 Lead contact

## Abstract

Hypothalamic neural circuits regulate instinctive behaviors such as food seeking, the fight/flight response, socialization, and maternal care. Here, we identified microdeletions on chromosome Xq23 disrupting the brain-expressed transient receptor potential (TRP) channel 5 (TRPC5). This family of channels detects sensory stimuli and converts them into electrical signals interpretable by the brain. Male *TRPC5* deletion carriers exhibited food seeking, obesity, anxiety, and autism, which were recapitulated in knockin male mice harboring a human loss-of-function *TRPC5* mutation. Women carrying *TRPC5* deletions had severe postpartum depression. As mothers, female knockin mice exhibited anhedonia and depression-like behavior with impaired care of offspring. Deletion of *Trpc5* from oxytocin neurons in the hypothalamic paraventricular nucleus caused obesity in both sexes and postpartum depressive behavior in females, while *Trpc5* overexpression in oxytocin neurons in knock-in mice reversed these phenotypes. We demonstrate that TRPC5 plays a pivotal role in mediating innate human behaviors fundamental to survival, including food seeking and maternal care.

## In brief

Disruption of TRPC5, a brain-expressed cation channel, causes obesity, maladaptive behavior, and postpartum depression in humans and mice. Acting on hypothalamic Pomc and oxytocin neurons, TRPC5 regulates instinctive behaviors such as feeding, arousal, social interaction, and maternal care, which are fundamental for survival.

## Graphical Abstract

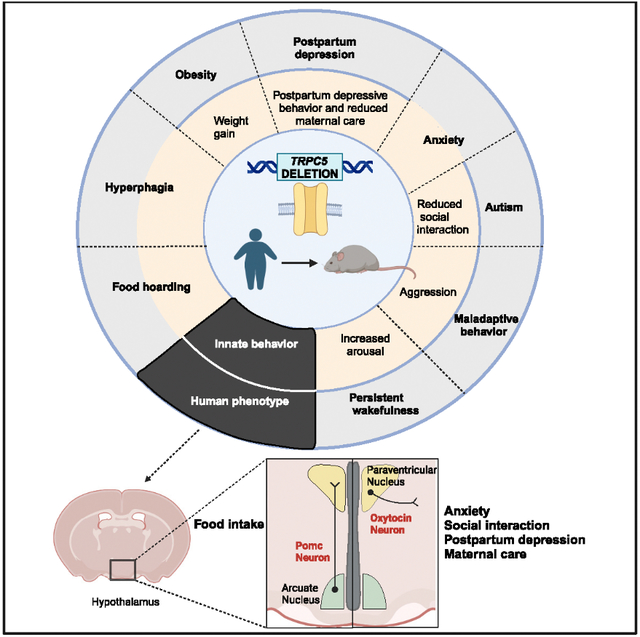

## INTRODUCTION

Innate or instinctive behaviors such as finding food, caring for offspring, self-preservation, and cooperation/socialization are genetically encoded responses that are critical for survival and reproductive success.^1,2^ Experiments in model organisms have shown that neurons within distinct nuclei of the hypothalamus regulate the initiation and maintenance of behaviors including feeding, sleep, aggression, and maternal care.^3–5^ These neuronal circuits sense changes in the internal state and external conditions and then select the most adaptive behavioral response (e.g., flight or fight)^6,7^ enabling variability or plasticity of hard-wired innate behaviors. To date, our understanding of the mechanisms by which internal and external sensory cues are integrated to coordinate innate behavior is limited, and the contribution of mechanisms studied in animals to human physiology and pathophysiology remains poorly understood.

Transient receptor potential (TRP) proteins are membrane-expressed calcium-permeable cation channels that transduce sensory stimuli into electrical signals that can be interpreted by the brain.^8,9^ TRP channels can be activated by biophysical (voltage, temperature, and pressure) and biochemical stimuli via G protein-coupled receptors (GPCRs), receptor tyrosine kinases, and depletion of intracellular calcium stores.^9–13^ These polymodal properties enable the detection and integration of environmental and endogenous sensory cues.^14,15^ For example, a specific TRP channel (TRPV1) detects heat and the taste of capsaicin (chili),^16^ while another (TRPM8) detects cold temperatures and the taste of menthol.^17^

Here, we focused on the brain-expressed TRP channel, transient receptor potential channel 5 (TRPC5), which is expressed on hypothalamic pro-opiomelanocortin (Pomc) neurons, which regulate energy homeostasis in response to leptin, insulin, and serotonin.^18,19^ Targeted disruption of *Trpc5* in the brain and in Pomc neurons causes obesity due to increased food intake and reduced energy expenditure in mice.^20^ We identified microdeletions on the X chromosome that disrupt *TRPC5* in two boys with intense food-seeking behavior, obesity, autism, anxiety, increased arousal, and maladaptive behavioral responses to sensory cues. Their mothers were heterozygous deletion carriers and had obesity, anxiety, and postpartum depression. To explore mechanisms underlying these phenotypes, we generated a knockin mouse model of a human loss-of-function (LoF) *TRPC5* mutation. Male knockin mice exhibited weight gain on a high-fat diet (HFD), anxiety, increased arousal, and reduced sociability. Female mutant dams (mothers) exhibited depression-like behavior postpartum, anhedonia, and impaired maternal-offspring interactions with a reduced suckling-induced rise in serum prolactin compared to wild-type (WT) littermate dams. These studies demonstrate remarkable fidelity of the phenotypes seen in TRPC5-deficient humans in a knockin mouse model and establish that TRPC5 regulates a spectrum of innate behaviors across mammalian species.

We found high levels of Trpc5 expression in oxytocin (OXT) neurons in the paraventricular nucleus of the hypothalamus (PVH) known to regulate energy homeostasis and the response to stress, emotion, and social behaviors, including mother-infant bonding.^21–23^ Deletion of *Trpc5* from PVH OXT neurons caused severe hyperphagic obesity, postpartum depressive behavior, and reduced maternal care, while Trpc5 overexpression in PVH OXT neurons in knockin mice reversed these phenotypes. Cumulatively, these studies establish that TRPC5 acts on distinct neuronal populations in the hypothalamus to regulate innate behaviors including feeding, anxiety (flight/fight/fear), socialization, and maternal care.

## RESULTS

### Obesity, anxiety, and maladaptive behavior in people with TRPC5 deficiency

**Figure 1.**
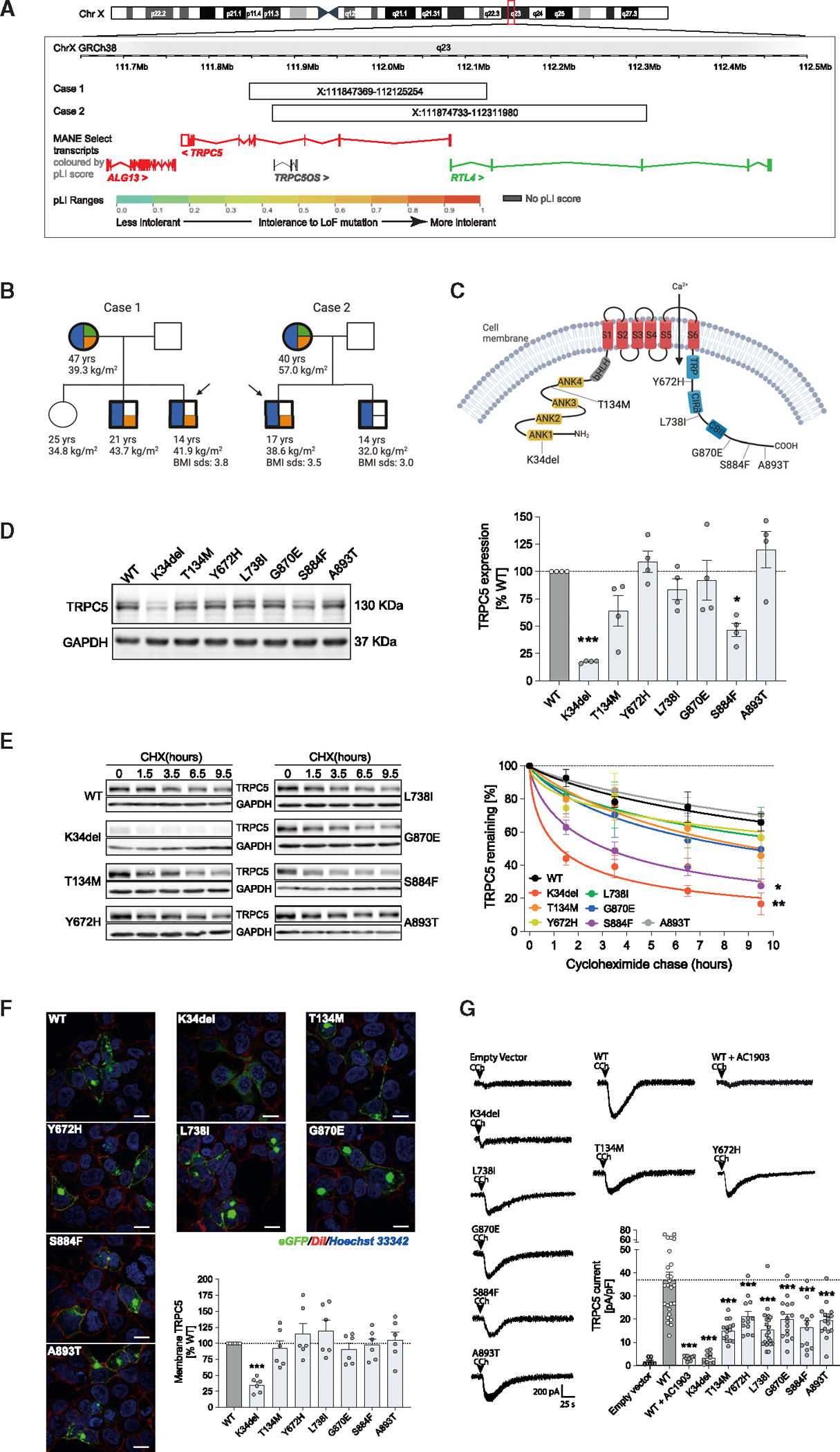
*TRPC5* variants identified in people with severe obesity (A) Deletions on the X chromosome disrupting *TRPC5* found in Cases 1 and 2; genes shown using a color code representing their tolerance to loss-of-function variants, pLI (loss intolerance probability). Coding and untranslated regions are shown in Figure S1A. (B) Co-segregation of deletions with obesity (blue), anxiety (orange), and postpartum depression (green) in families of Cases 1 and 2; males (squares), females (circles), and probands (arrows); age, BMI, and BMI standard deviation score (sds) for children under 18 years are indicated. (C) Rare *TRPC5* variants found in people with severe obesity shown on a schematic of the TRPC5 protein: NH2, amino-terminal domain; ANK, ankyrin domains 1–4; bHLH, basic helix loop helix; S1–S6, transmembrane helical domains; TRP, transient receptor potential domain; CIRB, calmodulin/inositol 1,4,5-triphosphate receptor binding domain; CBII, second calmodulin-binding domain; COOH, carboxy-terminal domain. (D–G) Functional characterization of TRPC5 variants. (D) Expression of WT/mutant TRPC5 in cells by western blotting (normalized to glyceraldehyde-3-phosphate dehydrogenase, (GAPDH) as a loading control). (E) Stability of WT/mutant TRPC5 in the cycloheximide (CHX) chase analysis. (F) Cell membrane localization of TRPC5; eGFP-tagged TRPC5 (green), DiI-stained cell membrane (red), and Hoechst33342-stained nucleus (blue). Scale bars, 20 μm. (G) TRPC5-mediated currents in cells transfected with empty vector, WT/mutant TRPC5 stimulated with the acetylcholine receptor agonist, carbachol (CCh); AC1903, TRPC5 inhibitor. Representative inward current traces and current density measured as ratio of peak current amplitude to cell membrane capacitance (pA/pF). Data expressed as % WT in (D) and (F); bars represent standard error of mean. *p* values were determined by unpaired t test with Welch’s correction; *p < 0.05, **p < 0.01, and ***p < 0.001. See also Tables S1–S3 and Figure S1.

Using comparative genomic hybridization, we identified microdeletions on chromosome Xq23 disrupting *TRPC5* in two boys with severe obesity from unrelated families of European ancestry in whom known genetic causes of obesity had been excluded (Figures 1A and S1A). Both probands had brothers who had severe obesity and carried *TRPC5* deletions, which were from their mothers who also had obesity (Figure 1B). Neither father had obesity or behavioral difficulties by report. These observations suggest that TRPC5 deficiency is an X-linked dominant obesity syndrome.

In childhood, probands displayed extreme food-seeking and hoarding behavior, hiding food for later consumption, in contrast to children with other monogenic obesity syndromes who seek food that is then rapidly consumed.^24^ Basal metabolic rate measured by indirect calorimetry was comparable to that predicted by age, sex, and body composition; there were no dysmorphic features nor any evidence of endocrine dysfunction (Table S1).

From age 2 years, both boys displayed outbursts of aggression and maladaptive behavior precipitated by sounds or smells that were perceived to be distressing. Both probands had features of autism and scored highly for autistic traits, thought problems, attention problems, anxiety, and/or depression (Figure S1B); they exhibited persistent wakefulness, often staying awake for 48 hours. Hemizygous males and heterozygous females with *TRPC5* deletions had a history of anxiety with panic attacks. Both mothers of the probands (who were heterozygous for *TRPC5* deletions) had early menarche (age 9–10 years), normal menstrual cycles, normal serum gonadotrophins, and prolactin levels. Both conceived without difficulty but experienced serious postpartum depression during the first 4 weeks following delivery.

To investigate whether rare variants in *TRPC5* may be identified in other people with severe obesity, we reviewed exome sequencing data from 984 people with severe childhood-onset obesity recruited to the Genetics of Obesity Study (GOOS; www.goos.org.uk).^25,26^ We did not find any microdeletions overlapping *TRPC5*. We did identify 7 rare (minor allele frequency, MAF<0.5%) coding variants that affect highly conserved residues and were either not found or rarely found in publicly available exomes (Figure 1C; Table S2). To investigate whether some variants affect function, we characterized the functional properties of normal (WT) and mutant forms of TRPC5 in transiently transfected HEK293 cells. Expression of K34del and S884F TRPC5 resulted in significantly lower protein levels compared with WT (Figure 1D). Using a cycloheximide chase assay, we found that K34del and S884F proteins displayed significantly accelerated degradation (Figure 1E), indicating impaired protein stability. Cell membrane expression of K34del was reduced, while other mutants exhibited normal levels of membrane expression (Figure 1F). Using electrophysiology, we demonstrated that HEK293 cells expressing WT TRPC5 display a robust inward current (Figures 1G and S1C), which was abolished by administration of a selective TRPC5 antagonist.^27^ In comparison to experiments with WT TRPC5, expression of all 7 TRPC5 mutants resulted in significantly reduced currents (Figures 1G and S1C), causing a LoF. Six out of seven variant carriers reported hyperphagia and food-seeking behavior, and three had a history of food hoarding (Table S2).

To investigate whether variants in *TRPC5* are associated with BMI in the general population, we examined ~450,000 individuals with exome sequence data from the UK Biobank study (Table S3). There were far fewer male carriers of *TRPC5* variants in UK Biobank (88 men compared with 281 women), possibly because people with severe obesity and/or complex behavioral phenotypes may be less likely to volunteer for research studies. In females, we found that *TRPC5* was the most strongly associated X-linked gene for BMI in the UK Biobank study (Figure S1D). The 369 individuals (88 men, 281 women) carrying heterozygous damaging mutations had an increased BMI relative to non-carriers (0.86 kg/m^2^ ± 0.23, *p* = 1.9 × 10^−4^; female odds ratio = 1.07 (1.02–1.13), *p* = 0.006). Stratified analyses found female carriers were on average heavier (1.13 ± 0.29 kg/m^2^, *p* = 9.3 × 10^−5^, Figure S1E), while no association was seen in the smaller number of male carriers (*p* = 0.52). These findings indicate that rare variants in *TRPC5* affect BMI in the population.

### Male knockin mice harboring a human LoF *TRPC5* mutation exhibit obesity, anxiety, and reduced sociability

**Figure 2.**
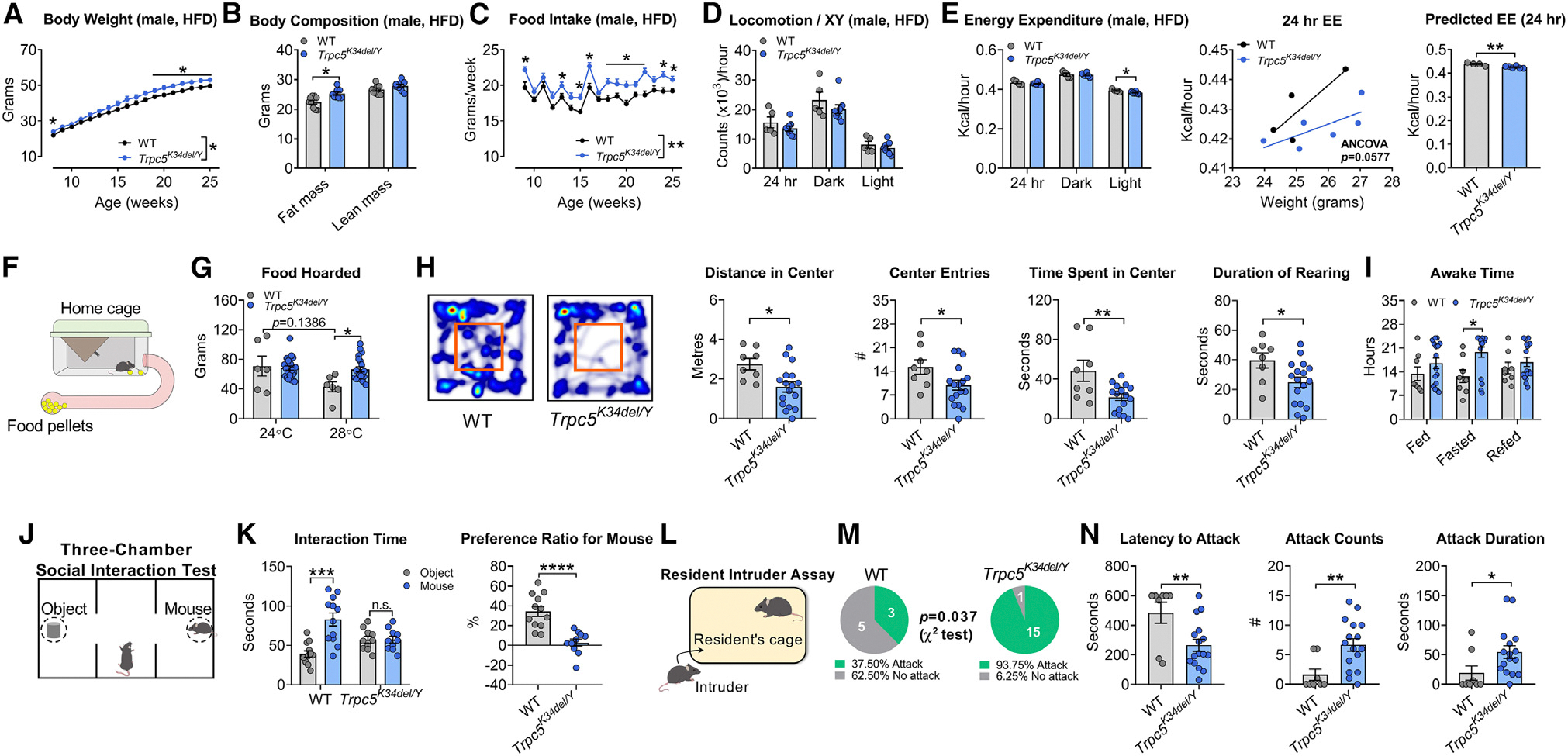
Metabolic and behavioral phenotype of male *Trpc5*^*K34del/Y*^ hemizygous mice (A–C) Experiments in male WT and *Trpc5*^*K34del/Y*^ hemizygous mice on a high-fat diet (HFD). Body weight (A), body composition (B), and weekly food intake (C) (*n* = 8–9 per group). (D) Locomotor activity (xy axis) during 24 h, dark and light cycles (*n* = 5–8 per group, 13 weeks of age). (E) Energy expenditure (EE) during 24 h, dark and light cycles, regression of EE with body mass, and predicted EE over 24 h based on 26 g body mass/mouse (*n* = 4–6 per group, 13 weeks of age). (F) Food-hoarding test. (G) Amount of food (grams) hoarded at 24°C or 28°C (*n* = 6–21 per group, 20 weeks of age). (H) Open field test: heatmap of movement, distance traveled in the center area, number (#) of center entries, time spent in the center, duration of rearing (*n* = 8–17 per group, 16 weeks of age). (I) Awake time during 24 h in fed, fasted, and refed conditions (*n* = 8–16 per group, 16 weeks of age). (J) Three-chamber test used to study social behavior. (K) Interaction time with object and mouse in chamber and preference ratio (mouse vs. object) (*n* = 10–12 per group, 12 weeks of age). (L) Resident-intruder assay. (M) % of mice that attacked intruders (*n* = 8–16 per group, 13 weeks of age). (N) Latency to attack, number (#) of attacks, and duration of attack (*n* = 8–16 per group, 13 weeks of age). Data presented as mean ± SEM, *p* value determined using 2-way ANOVA (A and C), unpaired t tests (B, D, E, G–I, K, and N), paired t tests (G), or chi-squared test (M). *p < 0.05, **p < 0.01, ***p < 0.001, and ****p < 0.0001. Overall difference between groups is indicated in the panel legend as appropriate. See also Figure S2.

To investigate whether mutations in *TRPC5* cause obesity and the behavioral phenotypes we observed, we generated a knockin mouse model of a human severe LoF mutation, K34del (*Trpc5*^*K34del*^ mouse line; Figure S2A). On a HFD, male *Trpc5*^*K34del/Y*^ mice exhibited significantly increased body weight and fat mass and hyperphagia (Figures 2A–2C). There was no change in physical activity but a significant decrease in energy expenditure in *Trpc5*^*K34del/Y*^ mice (Figures 2D and 2E). Male *Trpc5*^*K34del/Y*^ mice showed increased food-hoarding behavior when housed at 28°C ambient temperature (Figures 2F and 2G).

In the open field test, *Trpc5*^*K34del/Y*^ hemizygous male mice showed increased anxiety-like behavior: travel distance, number of entries into and time spent in the center, and duration of rearing in the open field arena were all reduced (Figures 2H and S2B–S2D). Using the home cage scan system, we observed that *Trpc5*^*K34del/Y*^ hemizygous male mice showed increased awake time specifically during a fasting period (Figure 2I). In a three-chamber social interaction test, compared with male WT mice, male *Trpc5*^*K34del/Y*^ hemizygous mice displayed significantly reduced interaction with another mouse (Figures 2J and 2K), demonstrating reduced sociability. In a resident-intruder test, a male “intruder” mouse was placed in the home cage of a “resident” mouse (male WT or *Trpc5*^*K34del/Y*^) that had been individually housed (Figure 2L). *Trpc5*^*K34del/Y*^ mice showed significantly increased attacks toward the intruder (93.75% in *Trpc5*^*K34del/Y*^ mice vs. 37.50% in WT mice, *p* = 0.037, χ^2^ test) with reduced latency, increased number, and duration of attacks (Figures 2M and 2N). Cumulatively, these experiments show that a human LoF mutation in *TRPC5* causes obesity, anxiety, reduced sociability, and increased aggressive behavior when modeled in male mice.

### *Trpc5* deficiency causes postpartum depressive behavior and impaired maternal care in female mice

**Figure 3.**
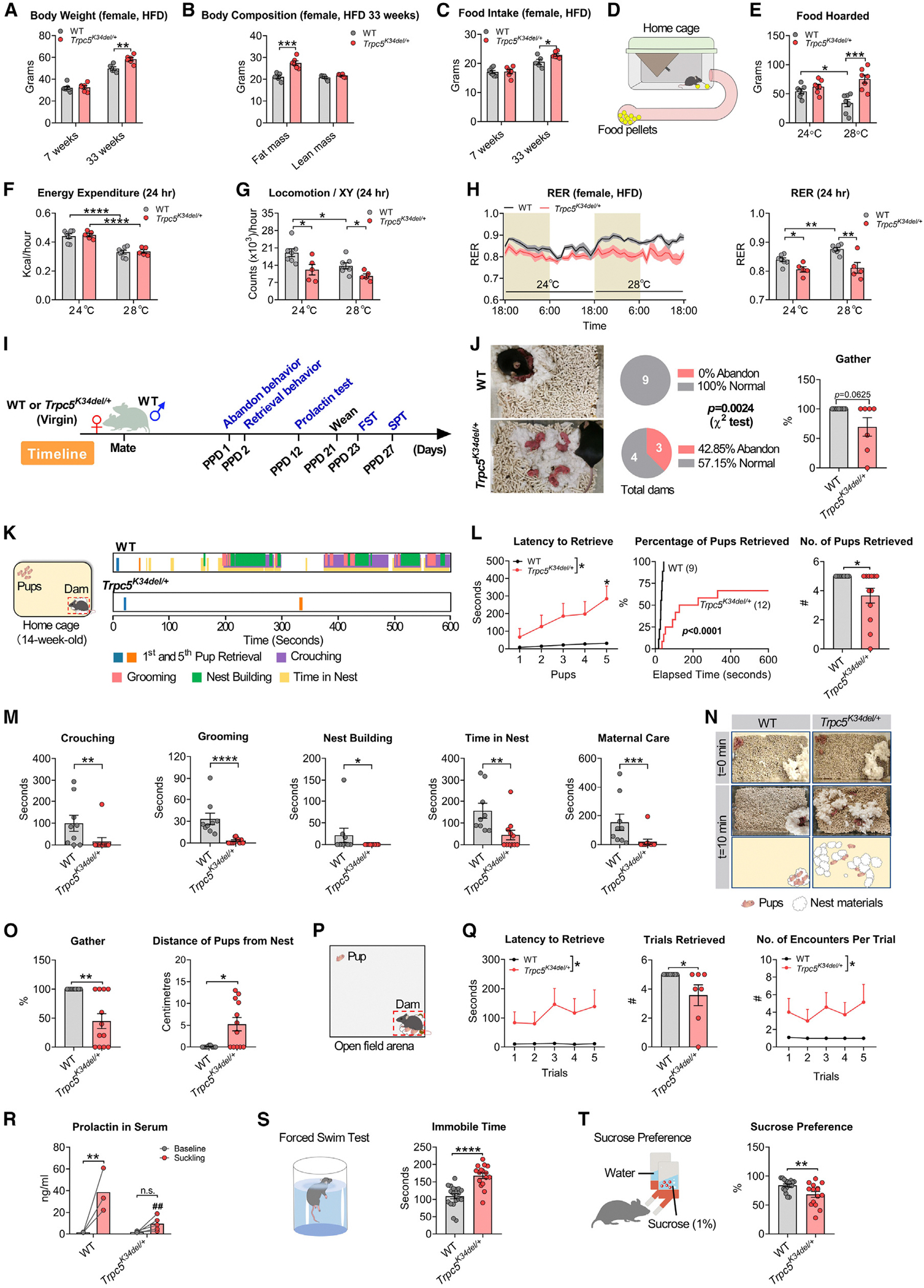
Metabolic and behavioral phenotype of female *Trpc5*^*K34del/+*^ heterozygous mice (A–H) Experiments in female WT and *Trpc5*^*K34del/+*^ heterozygous mice on a high-fat diet (HFD). Body weight (A), body composition (B), and food intake at 7 and 33 weeks (C) (*n* = 5–7 per group). (D) Food-hoarding test. (E) Amount of food (grams) hoarded at 24°C or 28°C (*n* = 7 per group, 20 weeks of age). Energy expenditure (F), locomotor activity (xy axis) (G), and respiratory exchange ratio (RER) (H) over 2 days (left) and during 24 h (right) at 24°C or 28°C (*n* = 5–7 per group, 20 weeks of age). (I) Protocol and timeline of tests of maternal behavior; PPD, postpartum day; FST, forced swim test; SPT, sucrose preference test. (J) Abandon behavior was observed on PPD1. Left: *Trpc5*^*K34del/+*^ dams failed to make a proper nest; pups were scattered randomly among the bedding; middle: % of dams that ignored and abandoned pups; right: % of dams gathered with pups in the nest (*n* = 7–9 per group, 14 weeks of age). (K) Left: maternal behavior assay in the home cage; right: sample behavior raster plot of WT and *Trpc5*^*K34del/+*^ dams. (L) Latency to retrieve pups, % of pups retrieved, number (#) of pups retrieved (*n* = 9–12 per group, 14 weeks of age). (M) Duration of crouching above pups, pup grooming, and nest-building behavior exhibited by dams, total time spent in nest, and duration of maternal care (*n* = 9–11 per group, 14 weeks of age). (N) Representative images at the beginning (t = 0 min) and end (t = 10 min) of the pup retrieval test (14 weeks of age). (O) % of pups gathered in the nest, distance of pups from the nest (*n* = 9–12 per group, 14 weeks of age). (P) Retrieval behavior in the open field arena. (Q) Latency to retrieve pups during 5 trials, number (#) of successfully retrieved trials among 5 trials, number of encounters with the pup in each trial (*n* = 7–9 per group, 14 weeks of age). (R) Serum prolactin at PPD 12 before and after suckling (*n* = 3–5 per group, 16 weeks of age). (S) Forced swim test, immobile time in forced swim test (*n* = 16–19 per group, 18 weeks of age). (T) Sucrose preference test, sucrose preference ratio (*n* = 15–18 per group, 18 weeks of age). Data presented as mean ± SEM, *p* value determined using 2-way ANOVA (H, L, and Q), unpaired t tests (A–C, E, F–H, and R–T), Mann-Whitney test (J, L, M, O, and Q), paired t tests (E–H and R), Kolmogorov-Smirnov test (L), or chi-squared test (J). *p < 0.05, **p < 0.01, ***p < 0.001, and ****p < 0.0001. Overall difference between groups is indicated in the panel legend as appropriate. See also Figure S2 and Video S1.

Female *Trpc5*^*K34del/+*^ heterozygous mice developed increased body weight and fat mass associated with increased food intake on a HFD (Figures 3A–3C). Female homozygous *Trpc5*^*K34del/K34del*^ mutants developed levels of obesity that were comparable to hemizygous males (Figures S2E and S2F). Female *Trpc5*^*K34del/+*^ mice also showed increased food hoarding when housed at 28°C ambient temperature (Figures 3D and 3E). We examined metabolic parameters in female WT vs. *Trpc5*^*K34del/+*^ mice in response to a 24°C–28°C environmental temperature shift. We observed an expected reduction in energy expenditure in both WT and *Trpc5*^*K34del/+*^ mice when the housing temperature was increased from 24°C to 28°C degrees (Figure 3F). While WT mice reduced their locomotor activity when the housing temperature was increased from 24°C to 28°C degrees, *Trpc5*^*K34del/+*^ mice showed reduced locomotor activity compared with WT mice regardless of the ambient temperature (Figure 3G). WT mice showed a significant increase in respiratory exchange rate (RER) when the housing temperature was increased from 24°C to 28°C (Figure 3H), consistent with the notion that at thermoneutrality, mice do not need to utilize as much fat to maintain energy homeostasis. Interestingly, while the RER of *Trpc5*^*K34del/+*^ mice was comparable to that of WT mice at 24°C, mutant mice did not increase their RER when switched to 28°C, resulting in a significantly lower RER compared with WT mice at thermoneutrality (Figure 3H). These data suggest that the *Trpc5*^*K34del*^ mutation impairs the adaptive response to changes in ambient temperature, which may contribute to the increased food-hoarding behavior observed in these mice at thermoneutrality.

To investigate the behavior of female *Trpc5*^*K34del/+*^ mice during the postpartum period, WT and *Trpc5*^*K34del/+*^ heterozygous female virgin mice were bred with experienced WT males (Figure 3I), resulting in successful pregnancies and comparable pup production, survival, and average body weight up to weaning (Figures S2G–S2I). On postpartum day (PPD) 1, significantly more *Trpc5*^*K34del/+*^ heterozygous dams showed abandon behavior compared with WT dams, with their pups found scattered within the bedding material (Figure 3J). We assessed the retrieval behavior of dams on PPD 2. *Trpc5*^*K34del/+*^ dams showed significantly increased latency to retrieve pups and reduced number of pup retrievals (Figures 3K and 3L; Video S1, first half). *Trpc5*^*K34del/+*^ dams also displayed significantly reduced crouching, grooming, nest building, and time in nest in keeping with a reduced level of maternal care (Figure 3M). Consequently, the percentage of pups gathered in the nest location was significantly reduced, and the distance to the nest location was significantly increased for pups cared for by *Trpc5*^*K34del/+*^ dams (Figures 3N and 3O). Similarly, in a pup retrieval test performed in a larger open field arena, *Trpc5*^*K34del/+*^ dams displayed significantly impaired retrieval behavior, demonstrated by increased latency to retrieve, decreased number of retrievals, and increased encounters per trial (Figures 3P and 3Q; Video S1, second half). *Trpc5*^*K34del/+*^ dams showed decreased travel distance and velocity (Figures S2J and S2K), suggesting a decreased motivation to approach pups. On PPD 12, we also detected severely impaired suckling-induced prolactin release in *Trpc5*^*K34del/+*^ dams, although their baseline prolactin levels (before suckling) were comparable to those of WT dams (Figure 3R). After weaning, *Trpc5*^*K34del/+*^ dams showed significantly increased immobility in a forced swim test and reduced interest in the rewarding properties of sucrose in a sucrose preference test, consistent with depression-like behavior and anhedonia (Figures 3S and 3T). Importantly, virgin *Trpc5*^*K34del/+*^ heterozygous female and *Trpc5*^*K34del/Y*^ hemizygous male mice did not exhibit depression-like behavior or anhedonia (Figures S2L and S2M). *Trpc5*^*K34del/+*^ heterozygous female mice also displayed increased anxiety-like behavior and increased awake time (Figures S2N–S2V).

The behavioral deficits observed in *Trpc5*^*K34del*^ mice suggest that activation of Trpc5 may improve these behaviors in WT mice. To test this, we used benzothiadiazine derivative (BTD), a Trpc5 activator with >15-fold selectivity for Trpc5 over other Trp channels.^28^ BTD significantly ameliorated anxiety-like behavior and improved social behavior in male WT mice and improved maternal behavior in female WT dams (Figure S3), providing additional evidence that these phenotypes are mediated by TRPC5.

### Genetic disruption of *Trpc5* impairs anorectic effects mediated by hypothalamic Pomc neurons

**Figure 4.**
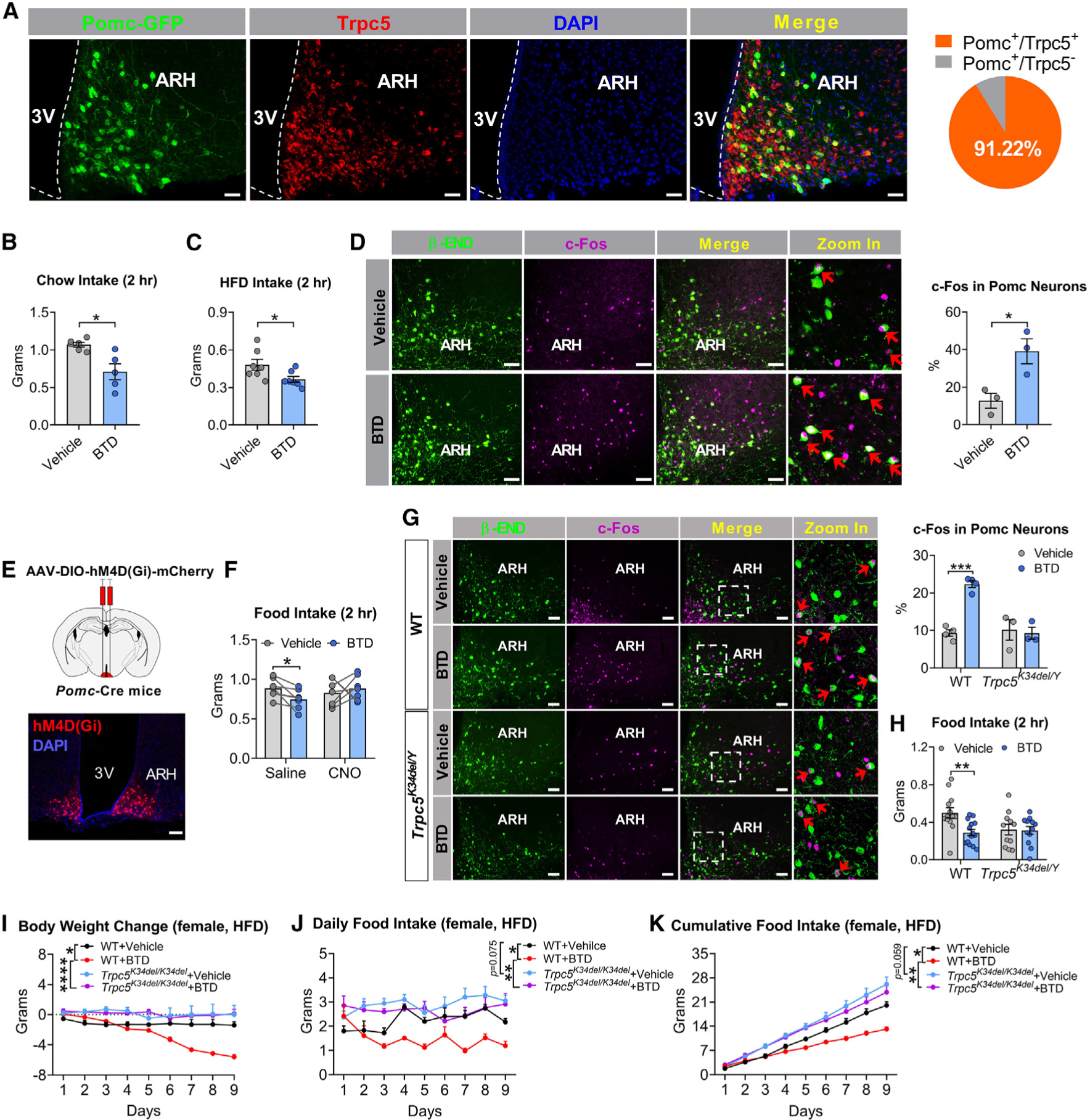
Trpc5 deficiency impairs anorectic effects mediated by Pomc neurons (A–C) (A) Representative images (left) and percentage (right) of ARH Pomc neurons (green) that express Trpc5 (red) in male WT mice (20 weeks of age). Scale bars, 25 μm; DAPI (4′, 6-diamidino-2-phenylindole) staining of nuclei; 3V is third ventricle. Chow intake (B) and HFD intake (C) within 2 h after injection of vehicle or a Trpc5 activator, benzothiadiazine derivative (BTD) in male WT mice (chow intake, *n* = 5–6 per group, 18 weeks of age; HFD intake, *n* = 7 per group, 28 weeks of age). (D) Immunoreactivity of β-endorphin (β-END; green), c-Fos (magenta), and merged images (yellow) and % of Pomc neurons (labeled by β-END) expressing c-Fos in vehicle or BTD-injected male WT mice (*n* = 3 per group, 16 weeks of age). Scale bars, 50 μm. (E) Upper: expression of hM4D(Gi) virus in Pomc neurons; lower: immunofluorescence images showing Pomc neurons expressing hM4D(Gi). Scale bars, 25 μm. (F) Effects of CNO co-injected with vehicle or BTD on food intake in male Pomc-Cre mice receiving inhibitory AAV8-hSyn-DIO-hM4D(Gi)-mCherry infection in the ARH (*n* = 7 per group, 20 weeks of age). (G) Immunoreactivity of β-END (green), c-Fos (magenta), and merged images (yellow) after injection of vehicle or BTD in male WT and *Trpc5*^*K34del/Y*^ mice (*n* = 3–4 per group, 12 weeks of age) and % Pomc neurons (labeled by β-END) expressing c-Fos (*n* = 3–4 per group, 12 weeks of age). Scale bars, 25 μm. (H) Food intake within 2 h after injection of vehicle or BTD in male WT and *Trpc5*^*K34del/Y*^ mice (*n* = 11–14 per group, 8 weeks of age). (I–K) Chronic injection of BTD in HFD-fed female WT and *Trpc5*^*K34del/K34del*^ homozygous females. Body weight change (I), daily food intake (J), and cumulative food intake (K) (*n* = 3–5 per group, 20 weeks of age). Data presented as mean ± SEM, *p* value determined using 2-way ANOVA (I–K), unpaired t tests (B–D, G, and H), or paired t tests (F). *p < 0.05, **p < 0.01, ***p < 0.001, and ****p < 0.0001. Overall difference between groups is indicated in the panel legend as appropriate. See also Figures S3 and S4.

Within the arcuate nucleus of the hypothalamus (ARH), we found that 91% of Pomc neurons co-express Trpc5 (Figure 4A). Previous studies have shown that Trpc5 depolarizes Pomc neurons, and targeted deletion of *Trpc5* from these neurons leads to hyperphagia and obesity.^20^ Here, we found that administration of BTD reduced both chow and HFD intake in WT mice (Figures 4B and 4C), without causing aversive effects measured using the kaolin intake test and the conditioned flavor avoidance test (Figures S4A–S4D). BTD administration increased c-Fos immunoreactivity in ARH Pomc neurons (Figure 4D) and chemogenetic inactivation (designer receptors exclusively activated by designer drugs [DREADD]; STAR Methods) of hM4Di-expressing Pomc neurons with clozapine N-oxide (CNO) blocked BTD-induced anorexia (Figures 4E, 4F, and S4E–S4G). Additionally, BTD significantly increased c-Fos in Pomc neurons and reduced food intake in male WT mice; these effects were reduced in male *Trpc5*^*K34del/Y*^ hemizygous mice (Figures 4G and 4H). In a chronic study, daily injections of BTD significantly reduced body weight and food intake of HFD-fed WT females; these effects were attenuated in HFD-fed *Trpc5*^*K34del/K34del*^ females (Figures 4I–4K). These results demonstrate that disruption of *Trpc5* in our knockin mouse model impairs the suppression of food intake mediated by Pomc neurons in the ARH.

We then examined the effects of leptin and lorcaserin (a serotonin 2C receptor agonist) on food intake in male WT vs. *Trpc5*^*K34del/Y*^ mice. Both leptin and lorcaserin significantly reduced food intake in WT mice, but these effects were blunted in *Trpc5*^*K34del/Y*^ mice (Figures S4H and S4I). Both leptin and lorcaserin significantly increased c-Fos expression in Pomc neurons in male WT mice, but these effects were blunted in *Trpc5*^*K34del/Y*^ mice (Figure S4J). Together, these results are consistent with previous reports^20,29^ that *Trpc5* in Pomc neurons mediates the anorectic effects of leptin and serotonin, effects that are markedly reduced in the knockin mouse model of a human LoF *TRPC5* mutation.

### Deletion of *Trpc5* from OXT neurons causes obesity

**Figure 5.**
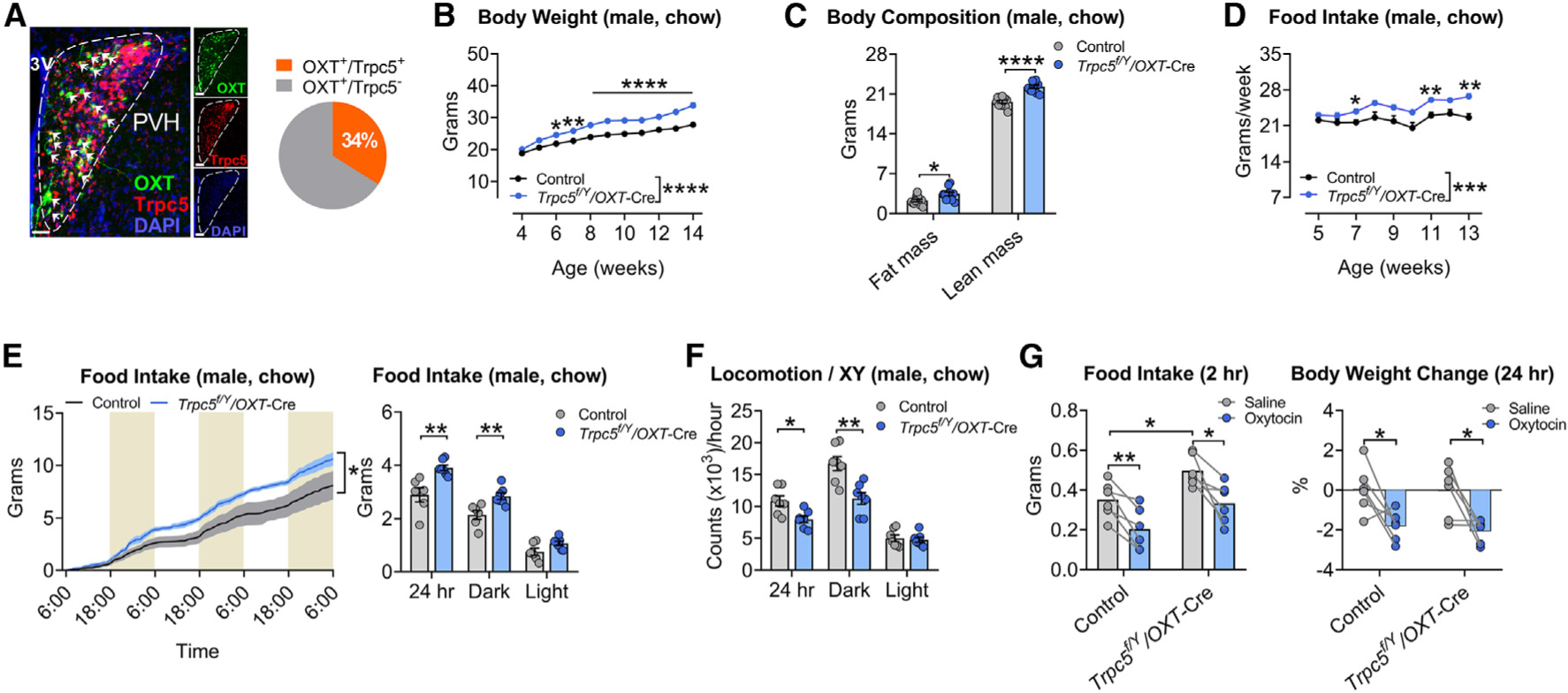
Metabolic effects of *Trpc5* deletion in OXT neurons (A–E) (A) Left: representative image showing Trpc5 (red) and OXT (green) expression in the PVH in male WT mice. Right: quantification of Trpc5 expression within PVH OXT neurons (*n* = 4 mice, 16 weeks of age). Scale bars, 50 μm. DAPI staining of nuclei; 3V is third ventricle. Experiments in male control and *Trpc5*^*f/Y*^*/OXT*-Cre mice on chow diet. Body weight (B), body composition (C), and weekly food intake (D) (*n* = 11 per group). (E) Cumulative food intake during 3 days (clock time shown) and during 24 h, dark and light cycles (*n* = 6–7 per group, 10 weeks of age). (F) Locomotor activity (xy axis) during 24 h, dark and light cycles (*n* = 7 per group, 10 weeks of age). (G) Food intake and body weight change after saline or OXT injection (*n* = 6 per group, 20 weeks of age). Data presented as mean ± SEM, *p* value determined using 2-way ANOVA (B, D, and E), unpaired t tests (C and E–G), or paired t tests (G). *p < 0.05, **p < 0.01, ***p < 0.001, and ****p < 0.0001). See also Figures S5 and S6.

We detected abundant Trpc5 expression in the PVH, co-localizing with 34% of OXT neurons (Figure 5A); Trpc5 did not co-localize with OXT in the supraoptic nuclei (SON) (Figure S5A). To investigate the contribution of Trpc5 on OXT neurons to the phenotypes we observed, we used the Cre-*loxP* approach to delete *Trpc5* in OXT neurons (*Trpc5*^*f/Y*^*/OXT*-Cre, Figure S5B). Deletion of *Trpc5* in OXT neurons in males and females produced marked hyperphagia, weight gain, and increased adiposity (when fed on chow) (Figures 5B–5D and S5C–S5G). These obesity-associated phenotypes were more severe than those seen in the humanized *Trpc5*^*K34del*^ mice. Experiments in TSE PhenoMaster metabolic cages confirmed increased food intake and decreased locomotor activity but no change in energy expenditure (using body mass as a covariate), indicating that hyperphagia is the major driver of obesity in mice lacking *Trpc5* in OXT neurons (Figures 5E, 5F, S5H, and S5I). These results indicate that in addition to Pomc neurons, Trpc5 acts on OXT neurons to regulate food intake and body weight. Supplementation of OXT in male and female *OXT-Trpc5-KO* mice reduced food intake and body weight to levels comparable to WT mice (Figures 5G, S5J, and S5K), suggesting that OXT analogs/receptor agonists could be effective in treating human obesity due to TRPC5 deficiency.

### Deletion of *Trpc5* from OXT neurons increases anxiety, impairs sociability, and causes postpartum depressive behavior in mice

**Figure 6.**
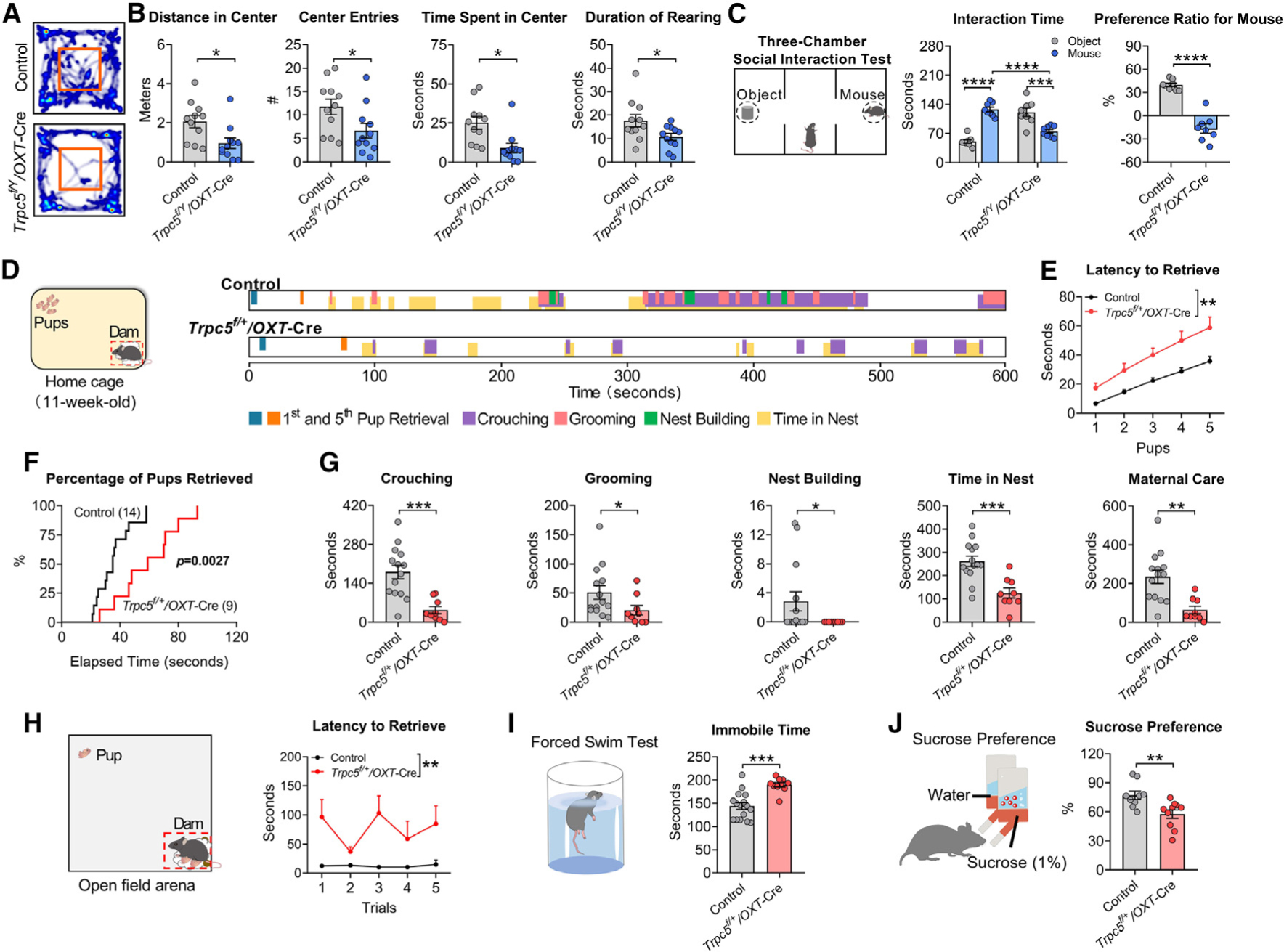
Behavioral effects of *Trpc5* deletion in OXT neurons (A–C) Experiments in male control and *Trpc5*^*f/Y*^*/OXT*-Cre mice. (A and B) Open field test: heatmap of movement, distance traveled in the center area, number (#) of center entries, time spent in the center, and duration of rearing (*n* = 11 per group, 11 weeks of age). (C) Three-chamber test used to study social behavior, interaction time with object and mouse in chamber, and preference ratio (mouse vs. object) (*n* = 8 per group, 16 weeks of age). (D) Maternal behavior assay in home cage, sample behavior raster plot of female control, and *Trpc5*^*f/+*^*/OXT*-Cre dams. (E–G) Maternal behavior assay in home cage. Latency to retrieve pups (E), percentage of pups retrieved (F), and duration of crouching above pups, pup grooming and nest-building behavior, total time spent in nest, and duration of maternal care (G) (*n* = 9–14 per group, 11 weeks of age). (H) Retrieval behavior in the open field arena and latency to retrieve pups during 5 trials (*n* = 9–13 per group, 11 weeks of age). (I) Forced swim test, immobile time in forced swim test (*n* = 10–16 per group, 15 weeks of age). (J) Sucrose preference test, sucrose preference (*n* = 9–10 per group, 15 weeks of age). Data presented as mean ± SEM, *p* value determined using 2-way ANOVA (E and H), unpaired t tests (B, C, I, and J), Mann-Whitney test (G), or Kolmogorov-Smirnov test (F). *p < 0.05, **p < 0.01, ***p < 0.001, and ****p < 0.0001. Overall difference between groups is indicated in the panel legend as appropriate. See also Figure S6.

We performed a series of behavioral tests in male *Trpc5*^*f/Y*^*/OXT*Cre and female *Trpc5*^*f/+*^*/OXT*-Cre mice and their control littermates. In virgin male and female mice, deletion of *Trpc5* from OXT neurons resulted in increased anxiety-like behavior (Figures 6A, 6B, and S6A–S6F). In addition, virgin male *Trpc5*^*f/Y*^*/OXT*-Cre mice showed reduced sociability (Figure 6C). The performance of virgin male *Trpc5*^*f/Y*^*/OXT*-Cre and female *Trpc5*^*f/+*^*/OXT*-Cre mice in tests of depression-like behavior was comparable to controls (Figures S6G–S6J).

To test whether Trpc5 in OXT neurons contributes to maternal behavior, we bred female *Trpc5*^*f/+*^*/OXT*-Cre mice and controls with experienced WT males, resulting in successful pregnancies and comparable pup production and average body weight up to weaning (Figures S6K–S6M). During lactation, female *Trpc5*^*f/+*^*/OXT*-Cre dams showed significantly reduced pup retrieval behavior and impairment of maternal care (Figures 6D–6H). After weaning, female *Trpc5*^*f/+*^*/OXT*-Cre dams showed increased depression-like behavior and reduced anhedonia (Figures 6I and 6J). In summary, deletion of *Trpc5* from OXT neurons recapitulates the behavioral deficits seen in the humanized *Trpc5*^*K34del*^ mice, including anxiety-like behavior, reduced sociability, impaired maternal behavior, and postpartum depression-like behavior.

### Restoration of Trpc5 in PVH OXT neurons reduces body weight, anxiety, and postpartum depressive behavior in *Trpc5*^*K34del*^ mice

**Figure 7.**
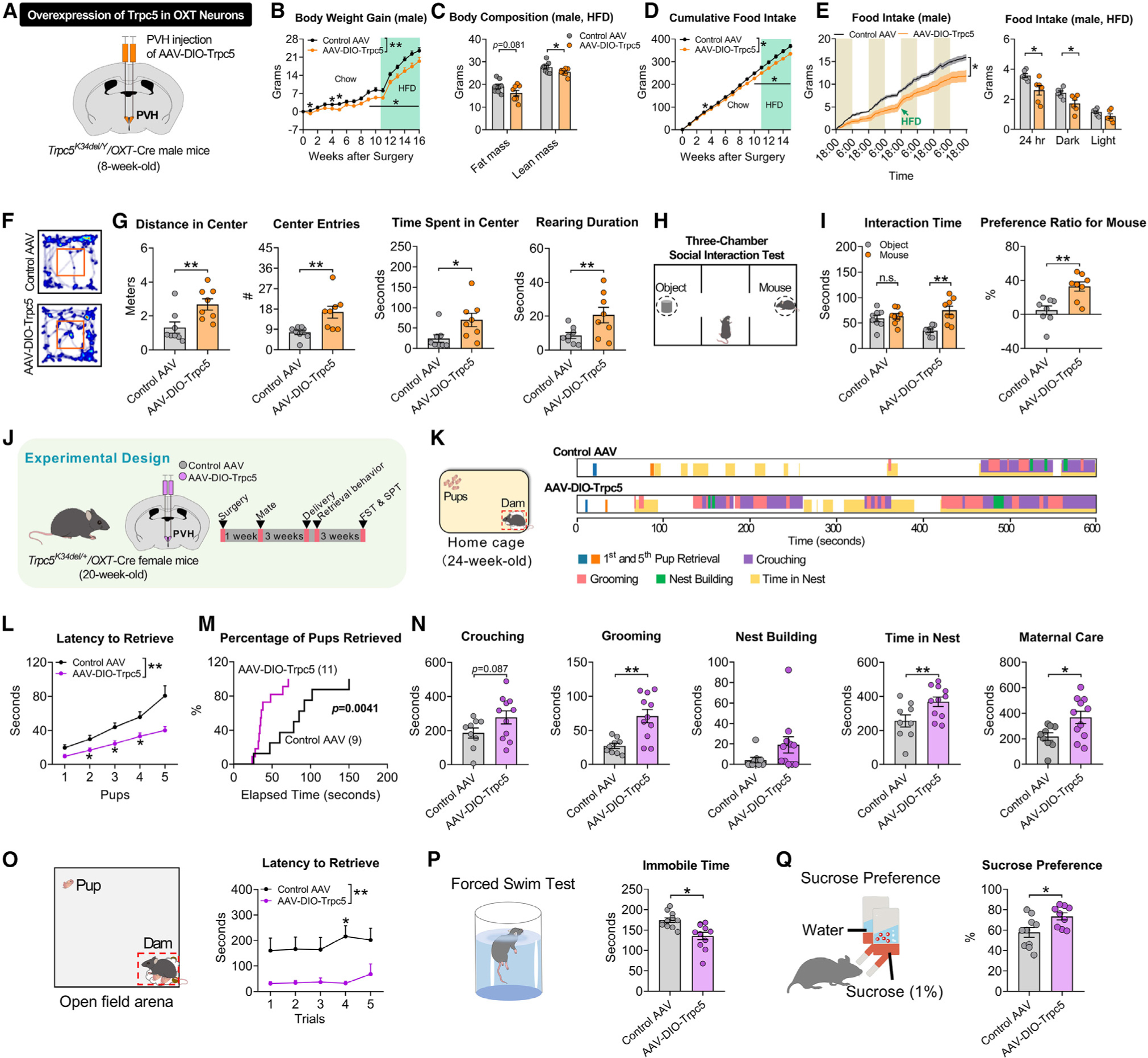
Restoration of Trpc5 in PVH OXT neurons reverses features of *Trpc5* deficiency (A–G) (A) Viral overexpression of Trpc5 selectively in PVH OXT neurons in male *Trpc5*^*K34del/Y*^*/OXT*-Cre mice. Body weight gain (B), body composition (C), cumulative food intake when fed chow and then high-fat diet (HFD) (D), food intake over 3 days and during 24 h, dark and light cycles (E) in male *Trpc5*^*K34del/Y*^*/OXT*-Cre mice receiving control adeno-associated virus (AAV) and AAV-DIO-Trpc5 (*n* = 6–8 per group, 22 weeks of age). Male *Trpc5*^*K34del/Y*^*/OXT*-Cre mice receiving control AAV and AAV-DIO-Trpc5 were studied (F–I). Open field test: heatmap of movement (F), distance traveled in the center area, number (#) of center entries, time spent in the center, duration of rearing (G) (*n* = 8 per group, 13 weeks of age). (H and I) Three-chamber social interaction test (H), interaction time with object and mouse in chamber and preference ratio (mouse vs. object) (I) (*n* = 8 per group, 13 weeks of age). (J) Experimental design to overexpress Trpc5 or GCaMP6m (control) selectively in PVH OXT neurons of virgin female *Trpc5*^*K34del/+*^*/OXT*-Cre mice, followed by the pup retrieval test, forced swim test (FST), and sucrose preference test (SPT). (K) Maternal behavior assay in home cage and sample behavior raster plot of female *Trpc5*^*K34del/+*^*/OXT*-Cre mice receiving control AAV and AAV-DIO-Trpc5. (L–N) Maternal behavior assay in home cage. Latency to retrieve pups (L), percentage of pups retrieved (M), duration of crouching above pups, pup grooming and nest-building behavior, total time spent in nest, and duration of maternal care (N) (*n* = 9–11 per group, 24 weeks of age). (O) Retrieval behavior in the open field arena and latency to retrieve pups during 5 trials (*n* = 7 per group, 24 weeks of age). (P) Forced swim test, immobile time in forced swim test (*n* = 11 per group, 28 weeks of age). (Q) Sucrose preference test, sucrose preference ratio (*n* = 9–10 per group, 28 weeks of age). Data presented as mean ± SEM, *p* value determined using 2-way ANOVA (B, D, E, L, and O), unpaired t tests (C, E, G, I, P, and Q), Mann-Whitney test (N) or Kolmogorov-Smirnov test (M). *p < 0.05 and **p < 0.01. Overall difference between groups is indicated in the panel legend as appropriate. See also Figure S7.

To examine the role of Trpc5 in PVH OXT neurons, we constructed a Cre-dependent AAV vector carrying the WT *Trpc5* (AAV-DIO-Trpc5) and validated that this virus overexpressed functional Trpc5 selectively in PVH OXT neurons when stereotaxically injected into the PVH of male and female *Trpc5*^*K34del*^*/OXT*-Cre mice (Figures S7A and S7B). We bilaterally injected AAV-DIO-Trpc5 into the PVH of male *Trpc5*^*K34del/Y*^*/OXT*-Cre mice to result in restoration of Trpc5 only in PVH OXT neurons in these mutant mice. As controls, another group of male *Trpc5*^*K34del/Y*^*/OXT*-Cre mutant mice received bilateral stereotaxic injections of a Cre-dependent AAV vector carrying GCaMP6m (a genetically encoded calcium sensor) into the PVH (Figure 7A). We found that the overexpression of Trpc5 only in PVH OXT neurons in *Trpc5*^*K34del/Y*^ mutant mice markedly reduced food intake, associated with a reduction in energy expenditure (probably reflecting a compensation), resulting in modest but significant decreases in body weight and fat mass (Figures 7B–7E, S7C, and S7D). In addition, overexpression of Trpc5 in PVH OXT neurons also improved anxiety-like and social behaviors in male *Trpc5*^*K34del/Y*^ mutant mice (Figures 7F–7I and S7E–S7G).

We then generated a female cohort of *Trpc5*^*K34del/+*^*/OXT*-Cre mice with or without overexpression of Trpc5 in PVH OXT neurons (Figure 7J). These mice were bred with experienced WT males, resulting in successful pregnancies and comparable pup production and average body weight up to weaning (Figures 7K and S7H–S7J). Overexpression of Trpc5 significantly improved pup retrieval behavior and indices of maternal care both in the home cage and the open field arena (Figures 7L–7O). After weaning, *Trpc5*^*K34del/+*^*/OXT*-Cre dams with Trpc5 overexpressed in PVH OXT neurons showed reduced depression-like behavior and reduced anhedonia (Figures 7P and 7Q). These data indicate that the postpartum depression-like behavior and impaired maternal behavior of Trpc5-deficient female mice are mediated by impaired function of PVH OXT neurons.

## DISCUSSION

We propose that disruption of TRPC5 in humans causes food-seeking and hoarding behavior, obesity, anxiety, and autism in males and postpartum depression in females. These phenotypes were recapitulated in male and female knockin mice harboring a human LoF mutation and in mice lacking Trpc5 expression in PVH OXT neurons. Viral-mediated overexpression of Trpc5 in OXT neurons in knockin mice reversed these phenotypes demonstrating the robustness of these findings and confirming a causal relationship. Cumulatively, we demonstrate that brain-expressed TRPC5 plays a critical role in mediating evolutionarily conserved innate behaviors in mammalian species.

### TRPC5 regulates food hoarding and energy intake

Using a pharmacological activator, we demonstrated that TRPC5 regulates the function of Pomc neurons in the ARH to reduce food intake and that this effect is impaired in a mouse model of human TRPC5 deficiency (*Trpc5*^*K34del*^), which exhibits hyperphagia. The hyperphagia is also partially mediated by impaired Trpc5 actions on OXT neurons in the PVH as deletion from this site caused hyperphagic obesity, which was reversed by OXT supplementation. Additionally, Trpc5 overexpression in PVH OXT neurons in *Trpc5*^*K34del*^ mice reduced food intake and body weight, further highlighting the role of PVH OXT neurons in mediating Trpc5 actions on feeding. Interestingly, the obesity seen in mice lacking Trpc5 in PVH OXT neurons was more severe than in the *Trpc5*^*K34del*^ knockin mouse model. There are several possible explanations for this observation. As mice expressing the severe LoF human *TRPC5* mutation express very little Trpc5 in the brain, the function of all neuronal populations expressing Trpc5 will be severely impaired. If Trpc5 is expressed in neurons whose normal function is to increase food intake, then deletion (*Trpc5*^*K34del*^ mice) would result in suppression of food intake, an effect which would counterbalance the weight-promoting effect of loss of Trpc5 in Pomc and OXT neurons. The degree of obesity seen in the *Trpc5*^*K34del*^ knockin mouse model is not as severe as one might expect given the human phenotype. Similar differences in the severity of obesity have been reported in mouse models of other human genetic obesity syndromes^30,31^ and may be explained by the access to and calorific value of food in the free-living vs. laboratory environment, differences in thermoregulation and environmental temperature, and inherent differences in lipid metabolism between species. Despite these limitations, the high fidelity with which human innate behaviors can be modeled in mice in this study enables the dissection of complex human phenotypes caused by TRPC5 deficiency.

Boys with TRPC5 deficiency displayed the highly unusual phenotype of food hoarding, a behavior that is not seen in other genetic obesity syndromes. Food hoarding is an adaptive strategy for animals living in an environment with an unpredictable food supply such as birds^32^ or those exposed to temperature extremes such as Siberian and Syrian hamsters.^33^ Although food hoarding is not routinely seen in laboratory rodents, we did observe increased food-hoarding behavior in Trpc5 mutant mice studied at 28°C (but not at 24°C). *Trpc5*^*K34del*^ mice did not increase their RER when switched to 28°C, indicating that the LoF mutation impairs the adaptive response to changes in ambient temperature, which may contribute to food-hoarding behavior at thermoneutrality.

Food deprivation increases food hoarding,^34^ effects which are blocked by systemic leptin administration in Siberian and Syrian hamsters.^35^ We hypothesize that a signal of unpredictable food availability (or proxy such as extreme environmental temperature) inhibits TRPC5, leading to food-hoarding behavior. When food is predictably available (laboratory animals and humans), there is no need to expend energy to hoard food, although acute food deprivation/fasting/leptin deficiency drives food seeking for immediate consumption.

### Role of TRPC5 in mediating postpartum depression and maternal care of offspring

From early pregnancy, female mice increase their nest-building behavior^36^ and spend several hours a day in the nest to care for the young after giving birth.^36^ Dams assume a crouching posture to enable suckling; they lick and groom pups extensively to keep them clean and retrieve the pups if they wander away from the nest. In female dams carrying a human LoF Trpc5 mutation and dams in whom Trpc5 was deleted or overexpressed in PVH OXT neurons, we demonstrate that this set of genetically encoded innate maternal behaviors is mediated by Trpc5 in OXT neurons.

Strikingly, the *Trpc5*^*K34del*^ mutation causes depressive behavior only in mouse dams but not in virgin females or males, highlighting a specific role for TRPC5 in the pathophysiology of postpartum depression. Overexpression of Trpc5 in PVH OXT neurons in *Trpc5*^*K34del/+*^ mutant dams ameliorated maternal depression-like behavior. Our findings align with studies in animals showing that impaired OXT signaling is associated with depression-like behavior^37^ and that PVN OXT neurons contribute to maternal care.^38^

In most mammals, the initiation of suckling depends on olfactory cues^39^; the action of suckling releases prolactin, which activates prolactin receptors to promote maternal nursing behavior.^40,41^ We found that the suckling-induced increase in serum prolactin was severely reduced in *Trpc5*^*K34del/+*^ mutant dams. A possible mechanistic explanation comes from work by Blum and colleagues^42^ who showed that Trpc5 maintains the infraslow membrane potential oscillations of hypothalamic dopaminergic neurons, which tonically inhibit prolactin secretion; deletion of Trpc5 from dopamine neurons leads to hypoprolactinemia. Of note, TRPC5-deficient humans and mutant mice retain their fertility (in contrast to mice lacking Trpc5 on dopaminergic neurons), which suggests a contribution from other neuronal populations.

### Trpc5 mediates socialization, anxiety, and arousal

It is likely that reduced TRPC5-mediated activation of OXT neurons contributes to reduced sociability in male knockin mice and autism in boys with *TRPC5* deletions. These findings are consistent with findings in obese mice and humans lacking *SIM1* (*Single-minded-1*) and *OTP* (*Orthopaedia*), transcription factors that regulate the development of the PVH, where hypothalamic OXT expression is markedly reduced and autism is a characteristic feature.^43–45^

We observed persistent anxiety in TRPC5-deficient people as well as in knockin mice. In rodents, Trpc5 is expressed in the amygdala, thalamus, somatosensory cortex, and other brain areas involved in mediating anxiety, characterized by excessive fear in response to objects or situations perceived as threats.^46^ Experiments in Trpc5 null mice suggest that TRPC5 may mediate innate fear and conditioned/learned fear responses.^47^ Additionally, serotonin 2C receptors modulate TRPC5 currents in hypothalamic Pomc neurons, and human LoF variants in the gene encoding the serotonin 2C receptor are associated with obesity and social anxiety.^48^ Experiments in rodents have informed the development of TRPC5 inhibitors as anxiolytics.^49,50^ Of note, the effects of Trpc5 disruption may in part be mediated by disruption of hetero-tetrameric complexes with TRPC4 and/or TRPC1.^51,52^ Our studies in human deletion carriers suggest that selective TRPC5 activators rather than inhibitors may be effective in the treatment of anxiety disorders.

A striking feature in the 2 male probands (and knockin mice) was persistent arousal. The spontaneous firing of hypothalamic orexin neurons maintains arousal; they are inhibited during sleep,^53^ and their auto-immune destruction causes narcolepsy. Previous studies in mice have shown that TRPC5 currents maintain orexin neurons in a depolarized state.^54^ Additionally, emotional cues lead to the activation of inhibitory GABAergic neurons in the central nucleus of the amygdala and induce cataplexy in orexin knockout mice.^55^ Understanding how TRPC5 regulates orexinergic circuits may reveal mechanisms modulating emotion/fear and states of heightened arousal.

### Clinical implications

These findings have implications for the diagnosis, treatment, and understanding of human physiology and disease. We recommend that *TRPC5* is included in diagnostic gene panels for severe childhood-onset obesity and for autism, which was a presenting feature in males. Intellectual disability and autism may also be present in males with larger X chromosome deletions encompassing *TRPC5*.^56^ The co-existence of hyperphagia and obesity in some children with intellectual disability and/or autism is often attributed simply to overeating or a lack of physical activity. However, there is increasing evidence that obesity, autism, intellectual disability, maladaptive behavior, and even hyperactivity can arise from the disruption of specific molecular and neurodevelopmental mechanisms.^43–45,57^

As the hyperphagia and obesity of TRPC5 deficiency are mediated by impaired activation of Pomc neurons, this disorder may be treatable with an MC4R agonist licensed for the treatment of genetic obesity syndromes.^58–60^ OXT receptor agonists or gene therapy to restore TRPC5 expression in specific areas of the hypothalamus are other potential therapeutic strategies.

Our findings are relevant for understanding postpartum depression, defined as a major depressive episode that occurs within 4 weeks post-delivery, which may persist for up to 1 year postpartum. It occurs in 10%–15% of pregnant women,^61^ and globally, postpartum depression remains a major cause of death by suicide in women.^62–64^ Mothers frequently experience difficulties caring for their infants,^62^ and adverse effects of maternal depression on infant behavior, emotional, and cognitive development can persist into childhood.^64^ The heritability of postpartum depression (30%–50%) is higher than for depression at other times.^65,66^ However, to date, no susceptibility loci have been identified,^67^ and the underlying mechanisms remain unknown. While OXT therapy has been trialed in women with postpartum depression,^68,69^ findings have been inconclusive. Our study provides direct causal evidence that reduced function of PVH OXT neurons can cause depression in the postpartum period and impairs maternal care. While TRPC5 deficiency is a very rare disorder, as with other monogenic diseases, its characterization reveals mechanisms that may be harnessed more broadly for therapy. We suggest it is now timely to investigate whether OXT agonists/analogs may benefit some women with postpartum depression in a randomized controlled trial.

In summary, we demonstrate that genetic disruption of TRPC5 in mice and humans leads to heightened arousal (wakefulness and anxiety), intense food seeking and food hoarding, and impacts on maternal behavior. These findings indicate that neuronal circuits expressing TRPC5 play an evolutionarily conserved role in mediating homeostatic and allostatic behavioral responses that are essential for survival.

### Limitations of the study

As the primary genetic finding in humans is based on the study of two families, the clinical characterization of additional families with rare deleterious mutations will be needed to establish penetrance and the mode of inheritance. Establishing whether rare variants found in population-based cohorts such as UK Biobank are pathogenic and, if so, estimating their penetrance will require further investigation as large-scale databanks and birth cohorts become available.^70^ Functional characterization of variants found in cases and controls will be needed as prediction algorithms are unlikely to be reliable given the complexity of molecular mechanisms that may be affected by missense variants. These studies are inherently challenging as people with severe phenotypes (potentially caused by penetrant variants) are less likely to volunteer for, and are therefore underrepresented in, such cohorts. Our studies in mice suggest that additional hypothalamic neurons express Trpc5. Activation or inhibition of these circuits may directly or indirectly influence the phenotypes we observed. Additional studies with targeted disruption and reactivation of Trpc5 in a wider range of neuronal populations will be needed to dissect its role in the integration of sensory stimuli to modulate innate behavior.

## STAR★METHODS

### RESOURCE AVAILABILITY

#### Lead contact

Further information and requests for resources and reagents should be directed to and will be fulfilled by the lead contact, Sadaf Farooqi (isf20@cam.ac.uk).

#### Materials availability

The data supporting the findings of this study are available within the main article and supplemental information. Reagents and source data for functional studies will be made available upon request to the lead contact Any additional information required to reanalyze the data reported in this paper is available from the lead contact upon request.

#### Data and code availability

- Data. GOOS WES data are accessible from the European Genome-phenome Archive-EGA:EGAS00001000124. Access to the UK Biobank genotype and phenotype data are open to all approved health researchers, accessible through https://www.ukbiobank.ac.uk. Requests for de-identified data relating to clinical studies may be addressed to the corresponding author (I.S.F.) Limitations on clinical data are designed to protect and respect patient and participant confidentiality.
- Code: this study did not generate any code.
- Additional information: this study did not generate any additional information.

### EXPERIMENTAL MODEL AND STUDY PARTICIPANT DETAILS

#### Human studies

All studies were approved by the Multi-Regional Ethics Committee and the Cambridge Local Research Ethics Committee (MREC 97/21 and REC number 03/103). Each subject (or their parent for those under 16 years) provided written informed consent; minors provided oral consent. All studies were conducted in accordance with the Declaration of Helsinki. UK people with severe obesity (BMI>3 SD above the mean for age and sex) of early onset (< 10 years) were recruited to the Genetics of Obesity Study (GOOS). Exome sequencing studies were undertaken as described previously.^25,26^

### METHOD DETAILS

#### Genetic studies

Both probands were investigated using comparative genomic hybridisation performed using the Affymetrix CytoScan 750K SNP genotyping array (analysed using Genome Reference Consortium Human genome build 37, GRCh37). The analysis was performed at a genome-wide resolution of 200 kb using Affymetrix Chromosome Analysis Suite (ChAS) software (Build 27). Overlapping deletions were identified using DECIPHER (https://deciphergenomics.org), a web-based platform correlating phenotype with genotype^72^: Case 1 (Decipher 281977); Case 2 (DECIPHER 351887). We excluded pathogenic variants in 79 genes whose disruption causes/is associated with obesity using a clinically approved test (https://www.preventiongenetics.com/sponsoredTesting/Rhythm/).

#### Detection of missense variants in *TRPC5*

*TRPC5* variants were detected in whole-exome sequencing (WES) data from 984 exomes from people with severe childhood-onset obesity recruited to the Genetics of Obesity Study (GOOS; www.goos.org.uk). Sequencing and variant-calling were previously described.^26^ Variants were annotated using Ensembl VEP v96 with GRCh37 human reference. Variants were filtered for consequence with respect to the canonical transcript ENST00000262839 (IMPACT=‘HIGH’ or ‘MODERATE’ or Consequence= ‘splice_region_variant’) and allele frequency in each queried population (minor allele frequency (MAF) <0.5% in 1000 Genomes Phase 3 continental populations,^73^ NHLBI-ESP^74^ and gnomAD exome populations. All variants were confirmed using Sanger sequencing in probands and family members. Sex and age information relating to the participants studied is included in Tables S1 and S2.

#### UK Biobank exomes and clinical phenotypes

To perform exome sequencing-based rare variant burden analyses, we queried population-level VCF files data for 454,787 individuals provided by the UKBB study via the UKBB Research Access Platform (https://ukbiobank.dnanexus.com/).^75^ For all downstream analyses, we excluded individuals with excess heterozygosity or autosomal variant missingness ≥ 5% on available genotyping array data or were not included in the subset of phased samples as defined in Bycroft et al.^76^

We defined a subset of ‘white European’ ancestry samples using a K-means clustering approach applied to the first four principal components calculated from genome-wide genotype array data. Individuals clustered into this group who self-identified by questionnaire as being of an ancestry other than white European were excluded.

Using bcftools^77^ multi-allelic variants were split and left-normalised, and all variants filtered using a missingness based approach. SNV genotypes with depth < 7 and genotype quality < 20 or InDel genotypes with a depth < 10 and genotype quality < 20 were set to missing. We also tested for an expected reference and alternate allele balance of 50% for heterozygous SNVs using a binomial test; SNV genotypes with a binomial test p. value ≤ 1×10^−3^ were set to missing. Following genotype filtering, variants with > 50% missing genotypes were excluded from further analysis. Variants were then annotated with the ENSEMBL Variant Effect Predictor (VEP) v104^78^ with the ‘everything’ flag and the LOFTEE plugin.^79^ For each variant we prioritised a single MANE v0.97 or VEP canonical ENSEMBL transcript and most damaging consequence as defined by VEP defaults. For the purposes of defining Protein Truncating Variants (PTVs), we grouped high-confidence (as defined by LOFTEE) stop gained, splice donor/acceptor and frameshift consequences. All variants were subsequently annotated using CADDv1.6.^80^

BMI for all participants was obtained from the UKBB data showcase (field 21001). After excluding individuals with missing data, 419,692 individuals with BMI measures remained for downstream analysis (191,864 males, 227,828 females). To assess the association between rare variant burden and BMI we implemented BOLT-LMM v2.3.5.^81^ As input to BOLT, we provided genotyping data,^76^ all WES variants passing quality control as defined above, and a set of dummy genotypes representing per-gene carrier status. For the latter, we collapsed variants across the *TRPC5* gene and defined carriers of variants as those with a qualifying “Damaging” variant, by combining high-confidence PTVs and missense variants with CADD > 25. For the women-only discovery analysis, genes with fewer than 100 carriers were excluded. BOLT-LMM was run with default settings and the ‘lmmInfOnly’ flag. All analyses were controlled for age, the first ten genetic ancestry principal components,^76^ WES batch, and sex when running sex-combined analyses.

#### Clinical studies

Probands carrying *TRPC5* deletions and variants and their families were invited to participate in research studies at the Wellcome-MRC Institute of Metabolic Science Translational Research Facility, Addenbrooke’s Hospital, Cambridge, UK. Height and BMI Standard Deviation Scores (SDS) were calculated using reference data for the UK.^82^ Quantitative measurements of behavior were obtained. The Autism Quotient is a 50-item questionnaire widely used in research and clinical practice.^83^ Questions assess five domains: social skills, attention switching, attention to detail, communication and imagination using a four-point rating scale in a forced choice format. The Achenbach System of Empirically Based Assessment (ASEBA) is widely used to assess adaptive and maladaptive behavior.^84^ Here, mothers of both probands completed the Child Behavior Checklist (CBCL/16–18-2001 version) which rates behavior and emotional problems over the preceding 6 months using a 3-point response format. CBCL problem scales and subscales assess symptoms of anxiety, depression and somatic complaints (associated with internalizing behaviors), social problems, thought problems, attention problems as well as externalizing behaviors (rule-breaking, impulsivity and aggression). CBCL total scores were derived using Assessment Data Manager software provided by www.aseba.org which converts raw scores into norm-referenced T-scores (T score < 65 normal; 65–69 borderline; ≥70 clinically significant).

#### Anthropometry, body composition, energy intake, and expenditure

Weight and height were measured barefoot in light clothing. Dual X-ray absorptiometry (DEXA) (DPX software; Lunar Corp) was used to determine body composition. Ad libitum energy intake was assessed using a 20 MJ meal of known macronutrient content (50% carbohydrate, 30% fat, 20% protein) after an overnight fast; food intake was expressed per kilogram of lean body mass as measured by DEXA to allow comparison between individuals. Basal metabolic rate and respiratory quotient were determined by indirect calorimetry after an overnight fast using an open circuit, ventilated, canopy measurement system (Europa Gas Exchange Monitor; NutrEn Technology Ltd.). Basal metabolic rate adjusted for body composition was compared to predicted metabolic rate based on standard age and sex specific equations, as previously described.^24^ Blood pressure was measured in the rested fasted state using automated brachial (DINAMAP, GE Healthcare) or wrist (OMRON Healthcare) monitors. Plasma glucose and insulin levels were measured using standard clinical assays.

#### Molecular studies

##### Cloning

The cDNA constructs used throughout the study were made using human wild-type *TRPC5* (NCBI NM_012471.2) ligated into pCDNA3.1(+) vector (Invitrogen) and C-terminally tagged with eGFP (GenScript; Clone ID: OHu18312C). *TRPC5* human mutations were introduced by site-directed mutagenesis using QuikChange II XL kit (Agilent Technologies, 200516) according to the manufacturer’s protocols and verified with Sanger sequencing.

##### Cell culture and transient transfection

HEK293 cells were purchased from Microbix Biosystems Inc. (Toronto, Ontario, Canada) and cultured in ATCC-formulated Eagle’s Minimum Essential Medium supplemented with 10% (v/v) of fetal bovine serum under 5% CO_2_ atmosphere at 37 °C humidified incubator. To construct TRPC5-transfected HEK 293 cells, constructs of pcDNA3.1(+1)-TRPC5-WT-eGFP and seven pcDNA3.1(+1)-TRPC5-mutation-eGFP were prepared, as described above. Cells were transiently transfected with TRPC5 plasmid DNA using Lipofectamine 3000 reagent (L3000015, Invitrogen) when reaching 70% confluence. The amount of plasmid DNA was adjusted according to the culture area of cells. Briefly, 500 ng and 2500 ng plasmid DNA were used for a well of 24-well and 6-well plate, respectively. Then the transfected cells were grown for 48 hours prior to experiments as outlined below.

##### Western blotting

To assess protein levels of TRPC5 in transfected HEK293 cells, Western blotting was performed. Briefly, 48 hours after the transfection, cells were harvested and lysed with RIPA buffer (J63306, Alfa Aesar) with a protease inhibitor cocktail (#11697498001, Millipore Sigma) and phosphatase inhibitors (#P0044, Millipore Sigma). Cell lysates were subsequently sonicated with 5 seconds pulse at 20% power using a probe sonicator and incubated on ice for 30 minutes. Lysates were centrifuged at 18000 g at 4 °C for 15 minutes, and the supernatants containing protein extracts were subjected to SDS-PAGE and immunoblot assay. The proteins were electrophoresed on a 10% SDS-polyacrylamide gel, then subsequently transferred to a polyvinylidene difluoride (PVDF) membrane. The membranes were probed with antibodies against TRPC5 (A10089, ABclonal) and GAPDH (G8795, Millipore Sigma) at 4°C overnight. The membranes were then incubated with either Alexa Fluor plus 680-conjugated secondary antibody (A32729, Invitrogen) or Alexa Fluor Plus 800-conjugated secondary antibody (A32735, Invitrogen) for 1 hour. Target bands were detected using a fluorescence scanner (Odyssey Infrared Imaging System, LI-COR Biotechnology), and were quantified using the ImageJ software. TRPC5 protein levels were normalized to GAPDH levels in each sample. WT TRPC5 expression levels were set as 100%. The results represent data from four independent experiments.

##### Cycloheximide chase assay

Cycloheximide chase assay was performed to assess protein stability. Cells were plated into 6-well plates. Forty-eight hours after the transfection, cells were incubated with medium containing 20 μg/mL of cycloheximide (C7698, Millipore Sigma) for 0, 1.5, 3.5, 6.5 and 9.5 h. At the end, cells were harvested for Western blotting as described above. Quantification of TRPC5 protein level normalized to GAPDH in each sample and then compared to time zero in each independent experiment, followed by a time-course nonlinear regression (sigmoidal with variable slopes) analysis of the data with a constrained fit [time zero] to constant 100, and presented for each condition (WT or mutant TRPC5). Results are from 3 independent experiments.

##### Confocal fluorescence imaging

For confocal imaging, TRPC5-transfected HEK293 cells were cultured on glass coverslips pre-coated with 2% gelatin. Prior to imaging, DiI dye (V22885, Invitrogen) was used to stain cell membranes as per manufacturer’s instructions. Afterwards, cells were washed with phosphate-buffered saline (PBS) for three times to remove excessive DiI dye and then fixed with 4% of paraformaldehyde in PBS for 15 minutes followed by PBS wash and nucleus staining with Hoechst 33342 dye (H3570, Invitrogen). Images were captured on an Olympus confocal microscope. Fluorescence intensity was quantified using ImageJ and six positive cells were selected for statistical analysis in each group. Results are from six cells in each group and from three independent experiments.

##### Electrophysiology in cultured cells

HEK293 cells were attached to circular glass coverslips and plated into 24-well plates. Cells were transiently transfected with 500 ng of plasmids for each group (WT or one of the 7 TRPC5 mutants), and whole-cell patch clamp recordings were performed 48 hours after transfection. Cells attached to circular glass coverslips were transferred to a chamber perfused with the standard bath solution containing (in mM): 140 NaCl, 5 CsCl, 2 CaCl_2_, 1 MgCl_2_, 10 glucose, and 10 HEPES (pH 7.4 with NaOH). GFP (+) cells were identified by using epifluorescence and IR-DIC imaging on an upright microscope (Eclipse FN-1, Nikon) equipped with a moveable stage (MP-285, Sutter Instrument). Borosilicate glass (Sutter instruments) pipettes (4–7 MΩ) were pulled with a horizontal pipette puller (P-1000, Sutter instruments) and were filled with artificial intracellular fluid (in mM): 110 CsCH_3_SO_3_, 25 CsCl, 2 MgCl_2_, 0.362 CaCl_2_, 1 EGTA, and 30 HEPES (pH 7.2 with CsOH). Recordings were made using a MultiClamp 700B amplifier (Axon Instrument), sampled using Digidata 1440A and analyzed offline with pClamp 10.3 software (Axon Instrument). Fast and slow capacitances as well as series resistance compensations were carefully adjusted. To record TRPC5 currents, the membrane potential was held at −60 mV in the voltage-clamp model. Another glass pipette filled with the acetylcholine receptor agonist, carbachol (CCh, 200 μM) was put at a 100 μm horizontal and 50 μm vertical distance from the recorded cell. A continuous current trace was recorded at the baseline for 0.5 minute before a 1 second puff delivery of CCh (200 μM) and for 3 minutes afterwards. The current-voltage relationships were recorded by 1s duration voltage ramps of −100 to +100 mV before and after CCh application. To ensure each recorded cell received the same amount of CCh, the puff pipette was always put at the same horizontal and vertical distance from the recorded cell. The puff strength was maintained at the same level by using a repeatable pressure pulse system (Picospritzer III, Parker). The selective TRPC5 blocker N-(2-Furanylmethyl)-1-(phenylmethyl)-1H-benzimidazol-2-amine (AC1903, #6766, Torics) was perfused at 50 μM via the bath solution in order to confirm whether the CCh-induced currents were mediated by TRPC5. To exclude the influence of the cell size on the current magnitude, all the currents were normalized to the cell membrane capacity. Results are from 6–26 cells in each group.

#### Mouse studies

##### Approval for studies in mice

Care of all animals and procedures were approved by the Baylor College of Medicine Institutional Animal Care and Use Committee. Age and developmental stage of experimental models are included in the relevant figure legend.

#### Generation of *Trpc5*^*K34del*^ mice

We used CRISPR-Cas9 gene editing to generate the knock-in mouse with the deletion of one amino acid residue (K34) in Trpc5 protein which was referred to as ***Trpc5*^*K34del*^**. The gene targeting was designed and performed by Genetically Engineered Rodent Models (GERM) Core at BCM. Briefly, the sgRNA (5′- TGAGACTGAGCTGTCTGCTG AGG -3′, reversed) and the single-stranded donor DNA (5’AGTTGATGTTGACATTGTAGTAGATCTCGGCCTCCTGCAGTGCCTGCTTCACTGTGGCATAGTCCCCCTTCTCCA CAGCACTAAGGAAGGCCTCTTCTGCAGACAGCTCAGTCTCAGCCCTCACGATTTGGAGGG-3’) were synthesized by IDT (Coralville, IA). The BCM GERM Core microinjected Cas9 (20 ng/μl), ssDNA (20 ng/μl), and sgRNA (20 ng/μl) into the pronuclei of 200 one-cell stage C57Bl/6J embryos as previously described.^30^ Founder animals (F_0_) were screened for the deletion by PCR amplification of tail DNA using the primer pairs: 5’-TGGCAACAGGGTCACATTGA and 5’-GGGCTTCCTGTAGCTAAGCA. The 534 bp PCR products were then digested with StuI which only recognized the site introduced by the deletion. After the digestion, 270 bp fragments could be detected from the mutant allele and was not cut for the WT allele. Three independent lines were sequenced for further confirmation of the deletion. One of these lines was crossed to C57Bl/6j to produce study cohorts.

#### Food intake, body weight, body composition, and TSE PhenoMaster metabolic cages

Both male and female WT and *Trpc5*^*K34del*^ littermates were singly housed from 4 weeks of age. All mice were fed ad libitum with a regular chow diet (5V5R-Advanced Protocol PicoLab Select Rodent 50 IF/6F, PicoLab) from weaning. A male cohort was switched to a high fat diet (HFD) (60% fat, #D12492i, Research Diets) from 8 weeks to 25 weeks of age; a female cohort was fed with a HFD from 13 weeks. Body weight and food intake were measured weekly after 7 and 33 weeks of HFD feeding. Body composition (fat mass and lean mass) was determined by quantitative magnetic resonance imaging during chow or HFD feeding at ages (or feeding weeks) specified in the figure legends. Another chow-fed male cohort was acclimated into the TSE PhenoMaster system at 13 weeks. In the TSE PhenoMaster cages, mice were maintained on a chow diet for the first 3 days, and then switched to HFD for the last 3 days, whilst energy expenditure and physical activity were continuously monitored. Energy expenditure data were analyzed with each animal’s body weight as a covariate using the online CalR tool.^85,86^

For the mouse cohorts with or without deletion of *Trpc5* in OXT neurons, both male and female mice were singly housed from 4 weeks of age. All mice were fed ad libitum with a regular chow diet from weaning. Body weight and food intake were manually recorded weekly. Mice were acclimated into the TSE PhenoMaster system at 10 weeks to measure energy expenditure, physical activity and food intake.

For the *Trpc5*^*K34del*^ mouse cohorts with or without Trpc5 overexpression in PVH OXT neurons, 8-week old *Trpc5*^*K34del/Y*^*/OXT*-Cre male mice were bilaterally injected with AAV-DIO-Trpc5 in the PVH followed by weekly body weight and food intake measurements. After 14 weeks, mice were acclimated into TSE PhenoMaster metabolic cages; they were maintained on a chow diet for the first 2 days while in the TSE cages, and then switched to HFD for the last 2 days. Meanwhile, energy expenditure, physical activity and food intake were continuously monitored as described above.

#### Validation of Trpc5 knockout in PVH OXT neurons

To validate *Trpc5* knockout in OXT neurons, *OXT*-Cre mice were crossed with Rosa26-LSL-tdTomato mice to generate male *OXT*-Cre/ Rosa26-LSL-tdTomato mice. Then, the latter was crossed with female *Trpc5*^*flox/+*^ mice to generate male *Trpc5*^*f/Y*^*/OXT*-Cre/Rosa26-LSL-tdTomato mice. Adult male *OXT*-Cre/Rosa26-LSL-tdTomato and *Trpc5*^*f/Y*^*/OXT*-Cre/Rosa26-LSL-tdTomato mice were anesthetized with isoflurane and quickly perfused with saline, followed by 10% formalin. Brains were immediately collected and postfixed in ice-cold 5% formalin overnight, after which they were cryoprotected in 30% sucrose in PBS. Coronal brain slices (30 μm) were prepared, and the Trpc5 staining was performed according to standard immunofluorescence protocols as described below.

#### Mouse behavioral tests

##### Maternal behaviors

The morning after birth, each neonatal pup’s location was noted in the home cage. The number of pups scattered in the home cage and the number of pups gathered in the nest area were recorded. The gather percentage was defined as the number of dams gathered with pups/all dams × 100%. Then, the total number of pups born, and the weight of the pups were assessed. Retrieval behavior was performed on postpartum day (PPD) 2 during the light cycle. Animals in their home cages were transported to the test room and were habituated to the environment for 30 minutes before testing. Retrieval behavior was tested by scattering five random biological pups (from each tested dam) at the corner away from the nest, then introducing their dams in the nest area in the home cage. The animals’ behavior was recorded for 10 minutes.

The behavior of the dams was scored using the following criteria: pup retrieval (picking up a pup with its mouth and moving it towards the nest site), crouching (nursing-like posture over at least 3 pups), grooming (sniffing and licking a pup), nest building (collecting and arranging nesting material and making a nest) and time in the nest. The time dams spent grooming, crouching or nest building after all the pups were retrieved, was reported as a “maternal care”. The latency time spent on retrieving was recorded as 10 minutes if the dam failed to retrieve the pups within 10 minutes. The percentage of all pups that were gathered and distance of pups to the nest were calculated by the end of the test. The heatmap for multiple behaviors during the 10 minutes was generated by Behavioral Observation Research Interactive Software (BORIS).

On the same day, the retrieval behavior was also tested in a larger open field arena (length × width × height: 42 × 42 × 30 cm). Dams were introduced into the test arena with a home base composed of some bedding material, nesting material, two food pellets, and two of its own biological pups in one corner. After 10 minutes of free exploration, the dam settled down in the home base and then one additional biological pup was introduced at the opposite corner of the home base. The path taken by dams was recorded by the camera. A total of 5 trials were tested, and each trial lasted 5 minutes or until the dam successfully retrieved the pup, whichever came first. The latency time spent before retrieving the pup was recorded (recorded as 5 minutes if the dam failed to retrieve the pup within 5 minutes). One encounter was defined as the dam directly making contact with the pup, and the number of encounters in each trial was recorded. Distance travelled and velocity were analyzed using the Noldus EthoVision XT software (Noldus, Leesburg, VA, USA).

##### Suckling and prolactin assay

To detect the level of prolactin in serum, we collected the blood from the tail of WT and *Trpc5*^*K34del/+*^ dams before and after suckling at PPD 12. Briefly, all dams were moved to a new cage with food and water. After 2 hours, a baseline blood sample was collected. Dams were returned to their previous cage for their pups to suckle for 1 hour and then blood collected again. Serum was obtained by centrifuging samples at 4°C and at 3000 rpm for 20 minutes. Prolactin level was assayed using ELISA (Thermo Fisher Scientific [Cat.# EMPRL]) according to manufacturer’s instructions.

##### Forced swim test

The forced swim test (FST) was performed in virgin males, virgin females, or in dams on PPD 23 (9 am-12 pm). In brief, one mouse was put in a glass cylinder (height: 50 cm, diameter: 20 cm) containing water at 23–25°C and depth of 20 cm, and mouse behavior was recorded for 6 minutes. The immobile time during the last 4 minutes was counted. At the end of the test, mice were removed from the cylinder, dried with a paper towel and returned to their home cages.

##### Sucrose preference test

The sucrose preference test (SPT) was performed in virgin males, virgin females or in dams on PPD 27 as previously described.^87^ In brief, mice were habituated with two bottles of water for 2 days. Mice were then water deprived for 24 hours and then were provided with a free choice of either drinking 1% sucrose solution or double distilled water for 2 hours during the dark cycle (6 pm-8 pm). Bottle positions were switched every 30 minutes. Sucrose preference was calculated by dividing the consumption of sucrose by the total consumption of both water and sucrose.

##### Open field test

The open field test (OFT) was performed in virgin males, virgin females (from 8 am-12 pm) in the open field arena (length × width × height: 42 × 42 × 30 cm). The lines divide the floor into sixteen evenly spaced squares (10.5 × 10.5 cm). The center consisted of four squares in the center of the device (21 × 21 cm). A mouse was first placed into the center of the area and allowed to explore for 5 minutes, and the paths of the animals was recorded by a video camera. Total distance travelled, velocity, distance travelled in the center, time spent in the center and entries into the center were analysed using the Noldus EthoVision XT (Noldus, Leesburg, VA, USA). In addition, the number of episodes of rearing and total duration of rearing were recorded manually. The arena was cleaned with 75% alcohol solution between different mice.

##### Three-chamber social interaction test

Rodents are naturally sociable and prefer to spend more time with another rodent rather than a novel object. The social interaction test used a three-chambered box with openings between chambers for the mouse to pass through. During the habituation, a subject mouse was placed in the behavior apparatus for 5 minutes with the chamber doors open. After the habituation session, the social preference test was performed. While the subject mouse was in the center chamber, a never-before-met intruder mouse was placed in a pencil cup in the right chamber. Meanwhile, a pencil cup containing a novel object was placed in the left chamber. The subject mouse was allowed to move freely for 10 minutes then the interaction time with each cup was manually recorded. The preference ratio was calculated by the following equations (RCT, right cup time; LCT, left cup time).

PreferenceRatio=(RCT-LCT)/(RCT+LCT)*100%

##### Resident-intruder test

Mice were singly housed in the resident cage for at least one week prior to testing. The cage remained uncleaned and unchanged for one week prior to testing, so that there were olfactory cues to enhance the resident mouse’s territoriality. We started the test by introducing an unfamiliar retired male breeder into the home cage in the afternoon, and 10 minutes later, the intruder was removed from the home cage. All behaviors were continuously recorded with a video camera during the entire 10-minute period. The recorded videos were analysed in a blinded fashion to measure the latency for the resident mouse to start attacks, the number of attacks and the time spent by the resident mouse in attacks.

##### Food hoarding test

Food hoarding cages consist of two main components: the home cage and the food storage chamber. The home cage is a standard mouse cage that is equipped with bedding, food, and water. The food storage chamber is a separate chamber connected to the home cage via a tube. It is used to store food that the mouse can access through the tube. The food storage chamber is fitted with a dispenser to make it easy for the mouse to access the food. Mice were singly housed in the food hoarding cages for 2 days for acclimatization. On day 3, food was removed from the home cage and 110 grams of regular chow diet was put in the food storage chamber. The amount of chow diet a mouse hoarded to the home cage during 24 hours was recorded. The food hoarding behavior was evaluated in an ambient temperature of 28°C and 24°C in different trials.

##### Viruses

We obtained the following AAV vectors from Addgene: AAV serotype 8 hSyn-DIO-hM4D(Gi)-mCherry (#44362, 2.9 × 10^13^ gc/ml), AAV serotype 9 hSyn-DIO-GCaMP6m.WPRE.SV40 (#100838, 2.7 × 10^13^ gc/ml) and AAV serotype 9 hSyn-DIO-GFP (#100043, 4.3 × 10^13^ gc/ml); AAV serotype 2-CMV7-DIO-saCas9 (#7122, 1 × 10^13^ gc/ml) from Vector Biolabs. To generate the construct of AAV-hSyn-DIO-Trpc5-flag, the cDNA construct used throughout the study was made using mouse wild-type Trpc5 (NCBI NM_009428.3) cloned into the backbone of AAV-hSyn1-DIO, and C-terminally tagged with flag tag. The AAV vector expressing wild-type Trpc5 was packaged by Baylor IDDRC Neuroconnectivity Core. All viruses were stored in aliquots at −80°C until use.

##### Stereotaxic injection

Mice were anaesthetized with isoflurane (4–5%) and maintained under isoflurane (1–1.5%) throughout the surgery. For detecting Trpc5 expression in Pomc neurons, 200 nl AAV-DIO-GFP was bilaterally injected into the ARH (anteroposterior (AP): −1.46 mm relative to bregma, mediolateral (ML): ± 0.15 mm, dorsoventral (DV): −5.9 mm) of *Pomc*-Cre male mice. For DREADD manipulation of Pomc neurons study, 200 nl AAV8-hSyn-DIO-hM4D(Gi)-mCherry was bilaterally injected into the ARH (AP: −1.46 mm, ML: ±0.15 mm, DV: −5.9 mm) of *Pomc*-Cre male mice (8 weeks old). For overexpression of Trpc5 in OXT neurons, 300 nl AAV-hSyn-DIO-GCaMP6m (Control group) or AAV-hSyn-DIO-Trpc5-flag (Rescue group) were bilaterally injected into the PVH (AP: −0.8 mm, ML: ±0.15 mm, DV: −4.82 mm) of adult virgin *Trpc5*^*K34del/Y*^*/OXT*-Cre male or *Trpc5*^*K34del/+*^*/OXT*-Cre female mice. For the validation of overexpression of Trpc5 in OXT neurons, 200 nl AAV-DIO-Cas9 (as controls) was injected into the left side and AAV-hSyn-DIO-Trpc5-flag was injected into the right side of PVH (AP: −0.8 mm, ML: ±0.15 mm, DV: −4.82 mm) in adult virgin *Trpc5*^*K34del/Y*^*/OXT*-Cre/Rosa26-LSL-tdTomato male or *Trpc5*^*K34del/+*^*/OXT*-Cre/Rosa26-LSL-tdTomato female mice. For detecting whether overexpression of TrpC5 rescues cellular efficiency in OXT neurons, *Trpc5*^*K34del/Y*^*/OXT*-Cre/Rosa26-LSL-tdTomato and *Trpc5*^*K34del/+*^*/OXT*-Cre/Rosa26-LSL-tdTomato virgin male or female mice received the AAV-DIO-Trpc5-flag injected into the right side of the PVH and the AAV-DIO-Cas9 (as controls) injected into the left side of the PVH (AP: −0.8 mm, ML: ±0.15 mm, DV: −4.82 mm).

##### Immunofluorescence

At the end of the study, mice were anaesthetized with inhaled isoflurane and quickly perfused with saline, followed by 10% formalin. Brains were immediately collected and postfixed in ice-cold 5% formalin overnight, after which they were cryoprotected in 30% sucrose in PBS. Coronal brain slices (30 μm) were prepared, and the standard immunofluorescence protocol was followed. Briefly, slices were washed in 0.1% PBST (PBS+0.1% Triton) three times, with an interval of 10 minutes between each wash. Then, the slices were blocked for 2 hours in 0.3% PBST with 5% normal goat serum. For detecting Trpc5 expression in Pomc neurons in WT male mice, the primary antibody against Trpc5 (1:1000 dilution; A10089; ABclonal) was added to the sections of ARH region. For detecting Trpc5 expression in OXT neurons in WT virgin male mice, primary antibodies against OXT (1:1000 dilution; MAB5296; Millipore; USA) and Trpc5 (1:500; A10089; ABclonal) were added to the sections of PVH region. To validate deletion of Trpc5 in OXT neurons, the primary antibody against Trpc5 (1:1000 dilution; A10089; ABclonal) was added to the sections from male OXT-Cre/Rosa26-LS-LtdTomato and Trpc5^f/Y^/OXT-Cre/Rosa26-LSL-tdTomato mice. To detect c-Fos expression in Pomc neurons after leptin, lorcaserin or BTD injections, primary antibodies against β-endorphin (1:10000; #H-02233; Phoenix Peptide) and c-Fos (1:1000 dilution; Ab208942; Abcam) were added to the sections of the ARH. To detect restoration of Trpc5 in OXT neurons in *Trpc5*^*K34del*^*/OXT*-Cre/Rosa26-LSL-tdTomato mice, the primary antibody against Trpc5 (1:1000 dilution; A10089; ABclonal) was added to the sections of PVH region. To detect restoration of Trpc5 in OXT neurons in response to BTD in *Trpc5*^*K34del*^*/OXT*-Cre/Rosa26-LSL-tdTomato mice, the primary antibody against c-Fos (1:1000 dilution; Ab208942; Abcam) was added to sections of PVH region. Then, the samples were incubated at 4°C on a shaker overnight. The following day, slices were rinsed with 0.1% PBST for 3 ×10 minutes and then incubated with goat anti-mouse 488 (1:500; #115–545-146; Jackson Immunoresearch) or goat anti-rabbit 488 (1:500; #111–545-144; Jackson Immunoresearch) secondary antibodies at room temperature for 2 hours while shaking. Slides were cover-slipped and analyzed using a fluorescence microscope. The numbers of each type of cell were counted manually.

##### Drug administration

All systemic treatments were delivered intraperitoneally (i.p). Leptin (#CYT-683; ProSpec) and lorcaserin (#A12598; Adooq Bioscience) were dissolved in saline. BTD (#6940; Tocris Bioscience) was dissolved in 10% dimethyl sulfoxide in saline. For conditioned flavor avoidance experiments, LiCl (L9650; Sigma) was dissolved in saline. CNO (#16882; Cayman; USA) was dissolved in saline and stored at −20°C. Oxytocin (#1910; Tocris Bioscience) was dissolved in saline and stored at −20°C. Saline or saline with 10% dimethyl sulfoxide were used as vehicle controls, respectively. For intraperitoneal administration, drugs were administered in a volume of 10 ml/kg mouse weight.

##### Effects of BTD injection on food intake in chow and HFD-fed mice

Male WT mice under chow (12 weeks of age) or HFD feeding (28 weeks of age fed HFD for 16 weeks) were fasted overnight (18:00 pm to 8:00 am). Then, these mice received i.p. injections of vehicle or BTD (10 mg/kg) at 8:30 am. Food was provided to the cages immediately after the injections and food intake was measured after 2 hours.

##### Effects of BTD on c-Fos expression in Pomc neurons

Briefly, male WT mice on chow (16 weeks of age) were fasted overnight (18:00 pm to 8:00 am) and then received i.p. injections of vehicle or BTD (10 mg/kg) at 8:30 am. Two hours later, the mice were anesthetized with inhaled isoflurane and quickly perfused with saline, followed by 10% formalin. Brain sections were collected, stained for β-endorphin and c-Fos followed by immunofluorescence.

##### Effects of BTD on kaolin intake in mice

Male WT mice on chow (12 weeks of age) received i.p. injection vehicle or BTD at the onset of dark phase. After that, kaolin pellets (K50001; Research Diets, USA) were provided and the amount consumed was measured after 2 hours.

##### Effects of BTD on conditioned flavor avoidance (CFA)

A two-bottle CTA test was performed as described previously.^88^ Briefly, mice are habituated to restricted water access for 90 minutes/day for 7 days, 2 hours after the onset of the light phase. Food is available ad libitum. Mice are assigned to one of three groups: (1) Vehicle (2) 10 mg/kg BTD (3) 95 mg/kg LiCl. CFA training consisted of two training days. On each training day, mice have access to two burettes containing the same flavor of Kool-Aid (cherry or grape) during the fluid access period of 90 minutes. Immediately after, each mouse received i.p. injection drug or saline as described above. The order of drug/saline exposure and paired flavors was counterbalanced on different days. Each training day was followed by a non-injection day where water was available during the 90 minutes fluid access period. Two days after the final training day, animals were provided with both flavors (one flavor per burette) during the 90-minute fluid access period. Fluid intake was measured after 45 mins to minimize side preference. Fluid intake was recorded again at 90 minutes.

##### Home cage scan test

Awake time duration was monitored using the Mouse Home Cage Scan (version 3.00.; Clever Sys, Inc).^89^ This system allows automated quantification of continuous ambulatory activities of mice throughout the 24-hour dark/light cycle. Mice were considered awake if there was movement within 80 seconds. Monitored results were retrieved as Microsoft Excel files for the whole testing period and summed up as counts per hour according to the indicated ZT. Briefly, the mice were acclimated to the Mouse Home Cage Scan cages for 3 days. After that, the system started to record the movement of mice for 3 consecutive days, with the first day with chow diet ad libitum, the second day with food removed, and the third day with food provided again.

##### DREADD manipulation of Pomc neurons

To determine if Pomc neurons mediate the anorexia effects of BTD, DREADD technology was used to inhibit Pomc neuron activity. At 8 weeks of age, inhibitory DREADD vector (AAV8-hSyn-DIO-hM4D(Gi)-mCherry) was stereotaxically injected into the ARH of male *Pomc*-Cre mice. After 3 weeks of recovery, Pomc-hM4Di mice were fasted overnight (6 pm-8 am) and received saline + vehicle injection on next day (8:30 am). Food was provided immediately, and food intake was monitored for 2 hours afterwards. For three further tests, the same cohort mice were subjected to the same procedure but received saline + BTD (10 mg/kg), 1 mg/kg CNO + vehicle and 1 mg/kg CNO + BTD (10 mg/kg). After each test, mice recovered at least 12 days before the next test. Food intake was recorded for 2 hours afterwards. After the study, all mice were perfused with 10% formalin and brain sections were collection. Brain sections were then mounted and mCherry signals were monitored under fluorescent microscope for validation of injection accuracy. Only those mice with mCherry signals exclusively in the ARH were included in analyses for feeding behavior. To validate that hM4Di-infected Pomc neurons can be inhibited by CNO, AAV8-hSyn-DIO-hM4D(Gi)-mCherry was stereotaxically delivered into the ARH of another cohort of *Pomc*-Cre mice as described above. Three weeks after virus infection, mice were sacrificed, and ARH-containing brain slices were prepared. Effects of CNO on resting membrane potential and firing rate of mCherry-labelled Pomc neurons were recording used electrophysiology.

##### Electrophysiology in brain slices

Electrophysiology recordings in mice were performed as previously described.^90^ Mice were deeply anesthetized with isoflurane and transcardially perfused with a modified ice-cold sucrose-based cutting solution (pH 7.3) containing 10 mM NaCl, 25 mM NaHCO_3_, 195 mM Sucrose, 5 mM Glucose, 2.5 mM KCl, 1.25 mM NaH_2_PO_4_, 2 mM Na-Pyruvate, 0.5 mM CaCl_2_, and 7 mM MgCl_2_, bubbled continuously with 95% O2 and 5% CO2. The mice were then decapitated, and the entire brain was removed and immediately submerged into the cutting solution. Coronal brain slices (220 μm) containing the ARH were cut with a Microm HM 650 V vibratome (Thermo Scientific) in oxygenated cutting solution. Slices were then incubated in oxygenated artificial CSF (aCSF; 126 mM NaCl, 2.5 mM KCl, 2.4 mM CaCl_2_, 1.2 mM NaH_2_PO_4_, 1.2 mM MgCl_2_, 11.1 mM glucose, and 21.4 mM NaHCO_3_, balanced with 95% O2/5% CO2, pH7.4) to recover ~25 minutes at 32°C and subsequently for 1 hour at room temperature before recording. Slices were transferred to a recording chamber and allowed to equilibrate for at least 10 minutes before recording. The slices were superfused at 32°C in oxygenated aCSF at a flow rate of 1.8–2 ml/min. mCherry-labeled neurons were visualized using epifluorescence and IR-DIC imaging on an upright microscope (Eclipse FN-1, Nikon) equipped with a movable stage (MP-285, Sutter Instrument). Patch pipettes with resistances of 3–5 MΩ were filled with intracellular solution (pH 7.3) containing 128 mM K-Gluconate, 10 mM KCl, 10 mM HEPES, 0.1 mM EGTA, 2 mM MgCl_2_, 0.05 mM Na-GTP and 4 mM Mg-ATP. Recordings were made using a MultiClamp 700B amplifier (Axon Instrument), sampled using Digidata 1440A and analyzed offline with pClamp 10.3 software (Axon Instruments). Series resistance was monitored during the recording, and the values were generally <10 MΩ and were not compensated. The liquid junction potential was +12.5 mV and was corrected after the experiment. Data were excluded if the series resistance increased dramatically during the experiment or without overshoot for action potential. Currents were amplified, filtered at 1 kHz, and digitized at 20 kHz. Current clamp was engaged to test neural firing frequency and resting membrane potential at the baseline or in response to CNO (10 μM, 5s puff). The puff strength was maintained at the same level using a repeatable pressure pulse system (Picospritzer III, Parker). Each neuron was recorded at least 1 minute baseline and only the neurons with stable baseline were used to test the CNO treatment. The values of resting membrane potential and firing frequency were averaged at baseline and in a 1-minute range containing the point with the maximal change in resting membrane potential after CNO puff.

##### Leptin, lorcaserin, and BTD-induced anorexia

Male WT and *Trpc5*^*K34del/Y*^ mice (16 weeks old for leptin and lorcaserin treatment, 8 weeks old for BTD treatment) were briefly fasted (15:00 to 17:30 pm). Then, these mice received i.p. injections of saline, leptin (5 mg/kg) and lorcaserin (3 mg/kg) or vehicle (10% dimethyl sulfoxide in saline) and BTD (10 mg/kg) at 17:30 pm. Food was provided to the cages at 18:00 pm and food intake was measured for 2 hours.

Male WT and *Trpc5*^*K34del/Y*^ mice (19 weeks old for leptin and lorcaserin treatment, 12 weeks old for BTD treatment) were briefly fasted for 2 hours (13:00 to 15:00 pm) to empty the stomach and then received i.p. injections of saline, leptin (5 mg/kg) and lorcaserin (3 mg/kg) or vehicle (10% dimethyl sulfoxide in saline) and BTD (10 mg/kg) at 15:00 pm. One hour later, mice were anesthetized with inhaled isoflurane and quickly perfused with saline, followed by 10% formalin. The brain sections were cut at 30 μm and collected into five consecutive series. c-Fos expression in Pomc neurons was quantified using immunofluorescence. Three or four mice were included in each group.

##### Effects of chronic BTD in body weight and food intake

20 weeks old WT and *Trpc5*^*K34del/K34del*^ female mice were fed with a HFD beginning at 4 weeks of age and were individually housed. Vehicle and BTD (10 mg/kg) were i.p. injected once daily. The body weight and food intake were successively measured until the end of the study.

##### Effects of oxytocin on food intake and body weight

For the acute oxytocin injection study, 20 week old male and female mice with or without deletion of *Trpc5* in OXT neurons were fasted from 15:00 pm to 17:30 pm. Then, initial body weight was measured followed by i.p. injections of saline and oxytocin (2 mg/kg) at 17:30 pm. Food was provided to the cages at 18:00 pm and food intake was measured for 2 hours; body weight was measured after 24 hours.

### QUANTIFICATION AND STATISTICAL ANALYSIS

Results were analyzed using GraphPad Prism 8. The difference between two groups was tested using a two-tailed unpaired t-test, Mann-Whitney test (if data were not normally distributed), chi-squared test, or Kolmogorov–Smirnov test. Paired t test was used for before and after treatments within one group. Two way ANOVA ± Sidak corrections were used for comparisons involving two variables ± multiple testing (e.g. genotype and time of treatments). The difference between three groups was tested using a one way ANOVA. All *p*-values are from 2-sided statistical tests. *p*<0.05 was considered statistically significant (*p<0.05, **p<0.01, ***p<0.001 and ****p<0.0001).

## Supplementary Material

supplemental

1

## Figures and Tables

**KEY RESOURCES TABLE:** 

REAGENT or RESOURCE	SOURCE	IDENTIFIER
Antibodies		
Anti-Rabbit Trpc5	ABclonal	Cat#A10089; RRID: AB_2757613
Anti-Mouse oxytocin	Millipore Sigma	Cat #MAB5296; RRID: AB_2157626
Anti-Mouse c-Fos	Abcam	Cat #ab208942; RRID: AB_2747772
Anti-Rabbit β-endorphin	Phoenix Peptide	Cat #H-022–33; RRID: AB_2314007
Anti-Mouse GAPDH	Millipore Sigma	Cat #G8795; RRID: AB_1078991
Goat Anti-Mouse 488 secondary antibody	Jackson ImmunoResearch	Cat #115–545-146; RRID: AB_2307324
Goat Anti-Rabbit 488 secondary antibody	Jackson ImmunoResearch	Cat #111–545-144; RRID: AB_2338052
Alexa fluor 800 goat anti-Mouse secondary antibody	Invitrogen	Cat #A32735; RRID: AB_2633284
Bacterial and virus strains		
AAV8-hSyn-DIO-hM4D(Gi)-mCherry	Addgene	Cat #44362
AAV9-hSyn-DIO-GCaMP6m.WPRE.SV40	Addgene	Cat #100838
AAV9-hSyn-DIO-GFP	Addgene	Cat #100043
AAV2-CMV7-DIO-saCas9	Vector Biolabs	Cat #7122
AAV-hSyn-DIO-Trpc5-flag	This paper	N/A
Chemicals, peptides, and recombinant proteins		
CCh	Tocris	Cat #2810
AC1903	Tocris	Cat #6766
BTD	Tocris	Cat #6940
Leptin	Protein Specialists	Cat #CYT-683
Lorcaserin	AdooQ Bioscience	Cat#A12598
Clozapine N-oxide (CNO)	Cayman	Cat #16882
Kool Aid Powder	Kraft foods	N/A
Sucrose	Millipore Sigma	Cat #S9378
DMSO	Avantor	Cat #BDH67001.400
10% Neutral Buffered Formalin	Avantor	Cat #16004–128
Lithium Chloride (LiCl)	Millipore Sigma	Cat #L9650
Oxytocin	Tocris	Cat #1910
Lipofectamine 3000 reagent	Invitrogen	Cat #L3000015
ATCC-formulated Eagle’s Minimum Essential Medium	ATCC	Cat #30–2003
Cycloheximide	Millipore Sigma	Cat #C7698
Critical commercial assays		
Prolactin (PRL) Mouse ELISA Kit	ThermoFisher Scientific	Cat #EMPRL
QuikChange II XL kit	Agilent Technologies	Cat #200516
Experimental models: Cell lines		
HEK293 cells	This paper	N/A
Experimental models: Organisms/strains		
Mouse (C57BL/6): *Trpc5^K34del^*	This paper	N/A
Mouse (C57BL/6): *Pomc*-Cre	Balthasar et al., 2004^71^	N/A
Mouse (C57BL/6): *OXT*-Cre	Jackson Laboratory	Stock No:024234
Mouse (C57BL/6): *Trpc5* loxP/loxP	This paper	N/A
Mouse (C57BL/6): ROSA-tdTomato reporter line	Jackson Laboratory	Stock No:007905
Recombinant DNA		
pcDNA3.1(+)-TRPC5-eGFP	GenScript	Clone ID: OHu18312C
Software and algorithms		
GraphPad Prism 8	https://www.graphpad.com/	https://www.graphpad.com/
Illustrator	https://www.adobe.com/	https://www.adobe.com/
Noldus EthoVision XT	https://www.noldus.com/	https://www.noldus.com/
Behavioral Observation Research Interactive Software (BORIS)	https://www.boris.unito.it/	https://www.boris.unito.it/
CalR	https://CalRapp.org	https://CalRapp.org
Other		
Standard lab diet (rodent)	PicoLab Rodent Diet	Cat #5V5R
High fat diet (rodent)	Research Diets	Cat#D12492i
Kaolin diet	Research Diets	Cat #K50001

## Highlights

- In humans, deletion of *TRPC5* causes obesity, anxiety, autism, and postpartum depression
- Male and female mice harboring a human *TRPC5* mutation recapitulate these phenotypes
- Effects are mediated by TRPC5 acting on hypothalamic Pomc and oxytocin neurons
- Brain-expressed TRPC5 regulates instinctive behaviors essential for survival
