# Supplementary material for: Loss of transient receptor potential channel 5 causes obesity and postpartum depression
Source: Cell. Author manuscript; Available in PMC 2025 Apr 1. (PMC11961024; doi:10.1016/j.cell.2024.06.001)
Supplement: supplemental [file NIHMS2063739-supplement-supplemental.zip › 1-s2.0-S009286742400641X-mmc1.pdf]

Cell, Volume *187*

Supplemental information

Loss of transient receptor potential channel 5 causes obesity and postpartum depression

Yongxiang Li, Tessa M. Cacciottolo, Na Yin, Yang He, Hesong Liu, Hailan Liu, Yuxue Yang, Elana Henning, Julia M. Keogh, Katherine Lawler, Edson Mendes de Oliveira, Eugene J. Gardner, Katherine A. Kentistou, Panayiotis Laouris, Rebecca Bounds, Ken K. Ong, John R.B. Perry, Inês Barroso, Longlong Tu, Jonathan C. Bean, Meng Yu, Kristine M. Conde, Mengjie Wang, Olivia Ginnard, Xing Fang, Lydia Tong, Junying Han, Tia Darwich, Kevin W. Williams, Yongjie Yang, Chunmei Wang, Shelagh Joss, Helen V. Firth, Yong Xu, and I. Sadaf Farooqi

Table S1: Clinical characteristics of probands and family members carrying deletions in *TRPC5*, related to Figure 1.

	**Case 1**			**Case 2**		
	**Proband**	**Brother**	**Mother**	**Proband**	**Brother**	**Mother**
**TRPC5**	Deletion	Deletion	Deletion/+	Deletion	Deletion	Deletion/+
**Sex**	Male	Male	Female	Male	Male	Female
**Age** (years)	14.4	21.7	47.1	17.2	14.1	40.9
**Height** (cm) **(sds)**	191 (3.1)	191	174	175 (-0.1)	166 (0.3)	164
**Weight** (kg)	153.2	160.5	119.7	118.6	88.8	153.6
**Body Mass Index** (kg/m^2) **(sds)**	41.9 (3.8)	43.7	39.3	38.6 (3.5)	32.2 (3.0)	56.9
**% Fat mass by DEXA**	46.9	47.2	-	47.7	54.8	59.0
**Food intake** (KJ/kg lean mass)	142	70	-	41	81	48
**Measured BMR** (MJ/day)	-	-	-	10.3	7.7	6.8
**Predicted BMR** (MJ/day)	-	-	-	9.6	8.0	10.1
**Respiratory Quotient**	-	-	-	1.1	0.9	1.1
**Systolic BP** (mmHg)	111	141	130*	123	111	119
**Diastolic BP** (mmHg)	65	91	75*	89	87	77
**Pulse** (bpm)	65	70	67	67	76	79
**Fasting glucose** (3.5 - 5.5 mmol/L)	4.4	4.0	5.0	4.8	4.5	5.0
**Fasting insulin** (0 – 60 pmol/L)	499	-	122	142	231	109

Abbreviations: BMR – basal metabolic rate, BP – blood pressure, mmHg – millimetres of mercury, bpm – beats per minute, DEXA – dual energy X-ray absorptiometry, sds – standard deviation score, * patient on antihypertensive medication. Data not available (-).

Table S2. Clinical characteristics of people with severe obesity carrying missense variants in *TRPC5*, related to Figures 1 and S1.

TRPC5 variant[1]	Y672H	Y672H	L738I	L738I	L738I	G870E	G870E
**Global minor allele frequency**	2.64×10^{-5}		4.93×10^{-4}			3.12×10^{-6}	
**HGVSp[2]**	p.Tyr672His		p.Leu738Ile			p.Gly870Glu	
**Coding sequence[2]**	c.2014T>C		c.2212C>A			c.2609G>A	
**Age** (years)	30.3	62.9	21.4	24.9	14.0	25.2	56.7
**Sex**	M	F	F	M	F	M	F
**Hyperphagia**	Yes	Yes	Yes	No	Yes	Yes	Yes
**Food hoarding**	Yes	No	Yes	No	No	Yes	No
**Asthma**	Childhood	Childhood	No	Yes	Yes	Childhood	Yes
**Difficulty sleeping**	No	Difficulty falling asleep	No	No	No	No	No
**Autism Spectrum Quotient**	13	21	23	17	20	15	6
**Other behaviors**	Risk taking	No	Emotional lability	No	Emotion al lability	Anxiety, depression	Post-natal depressi on Alcohol misuse
**Height (sds)** (cm)	171	157	180	187	157 (-0.3)	180	158
**Weight** (kg)	176.8	102.9	159.6	184.5	83.8	212.8	89.4
**Body Mass Index** (kg/m^2) **(sds)**	60.5	41.5	49.1	52.9	33.9 (3.1)	65.5	35.6
**% Fat mass by DEXA**	48.2	49.3	56.4	51.5	46.8	-	47.4
**Food intake** (KJ/kg lean mass)	52.1	19.1	-	37.8	-	-	82.4
**Measured BMR** (MJ/day)	-	6.8	10.5	10.6	7.3	-	5.7
**Predicted BMR** (MJ/day)	12.5	7.7	10.4	12.9	6.9	14.3	7.1
**Respiratory Quotient**	0.8	0.8	0.8	0.8	1.0	0.8	1

**Systolic Blood Pressure** (<140 mmHg)	162	142	126	129	111	141	141
**Diastolic Blood Pressure** (60 – 90 mmHg)	108	88	79	60	67	73	89
**Pulse** (60 – 100 bpm)	71	69	71	69	83	67	69
**Puberty onset** (age in years)	16	12	14	13	12	15	12
**Fasting glucose** (3.5 – 5.5 mmol/L)	4.9	4.4	4.3	4.2	-	4.7	4.6
**Fasting insulin** (0 – 60 pmol/L)	43	78	144	247	-	113	29
**Acanthosis nigricans**	No	Yes	Yes	No	Yes	No	No

[1]Global Minor Allele Frequency (MAF) for *TRPC5* variants identified in GOOS obtained from gnomAD v4 (https://gnomad.broadinstitute.org/). The other variants identified in GOOS and studied in cells but not in physiological studies were: K34del (c.100-102del; p.Lys34del; MAF: 0), T134M (c.401C>T; p.Thr134Met; MAF: 1.2×10^{-4}), S884F (c.2651 C>T; p.Ser884Phe; MAF: 1.06×10^{-4}), A893T (c.2677G>A; p.Ala893Thr; MAF: 1.02×10^{-4}). [2]Missense variants with respect to transcripts ENST00000262839.3 / NM_012471.3. All samples and measurements were taken in the rested, fasted state. Data not available is indicated (-). Abbreviations: sds, standard deviation scores; DEXA, dual energy X-ray absorptiometry; BMR, basal metabolic rate; bpm, beats per minute; HGVSp, Human Genome Variation Society protein sequence nomenclature; mmHg, millimetres of mercury.

Table S3. Single-variant analysis of body mass index for UK Biobank carriers of *TRPC5* variants found in GOOS, related to Figure S1.

			SEX COMBINED				FEMALE ONLY			
**Coding sequence variant[1]**	**HGVSp[1]**	**UK Biobank Variant[2] (GRCh38/hg38)**	**BETA**	**SE**	**P**	**Carrier N**	**BETA**	**SE**	**P**	**Carrier N**
c.2677G>A	p.Ala893Thr	X:111776558:C:T	0.2198	0.3231	0.500	131	0.2300	0.5596	0.6800	75
c.2651C>T	p.Ser884Phe	X:111776584:G:A	0.2435	0.3341	0.470	129	0.4712	0.5320	0.3800	83
c.2609G>A	p.Gly870Glu	X:111776626:C:T	4.9999	3.1242	0.110	2	5.2901	3.4260	0.1200	2
c.2212C>A	p.Leu738Ile	X:111779005:G:T	-0.0203	0.1519	0.890	586	-0.4341	0.2715	0.1100	317
c.2014T>C	p.Tyr672His	X:111782021:A:G	0.3340	0.6127	0.590	34	1.0047	1.2113	0.4100	16
c.401C>T	p.Thr134Met	X:111912790:G:A	0.7955	0.3132	0.011	135	1.0489	0.5751	0.0680	71
c.100_102del	p.Lys34del	-	NA	NA	NA	NA	NA	NA	NA	NA

We identified 6/7 of the variants highlighted in GOOS in UK Biobank. The number of carriers of these variants in UK Biobank ranges from no carriers (K34del) and ultra-rare (G870E: N=2 carriers, both female, BMI=29 and 34 kg/m^2) to low-frequency alleles as expected for X-linked genes that are under significant constraint (probability of being loss of function intolerant [Pli]=1)[68]. As expected, given their rarity, none of these individually showed a statistically significant association with BMI, with the exception of T134M in a sex-combined model (P=0.01). BETA, effect size estimate on BMI in kg/m^2; SE, standard error of BETA. P two-sided from BOLT-LMM, using a linear-mixed model. [1]Variant consequences with respect to transcripts ENST00000262839.3 / NM_012471.3. [2]UK Biobank variant identifiers shown as Chromosome: Position (GRCh38/hg38): REF:ALT (reference: alternate allele).
