# Supplementary material for: Loss of transient receptor potential channel 5 causes obesity and postpartum depression
Source: Cell. Author manuscript; Available in PMC 2025 Apr 1. (PMC11961024; doi:10.1016/j.cell.2024.06.001)
Supplement: 1 [file NIHMS2063739-supplement-1.pdf]

CellPress
OPEN ACCESS

Supplemental figures

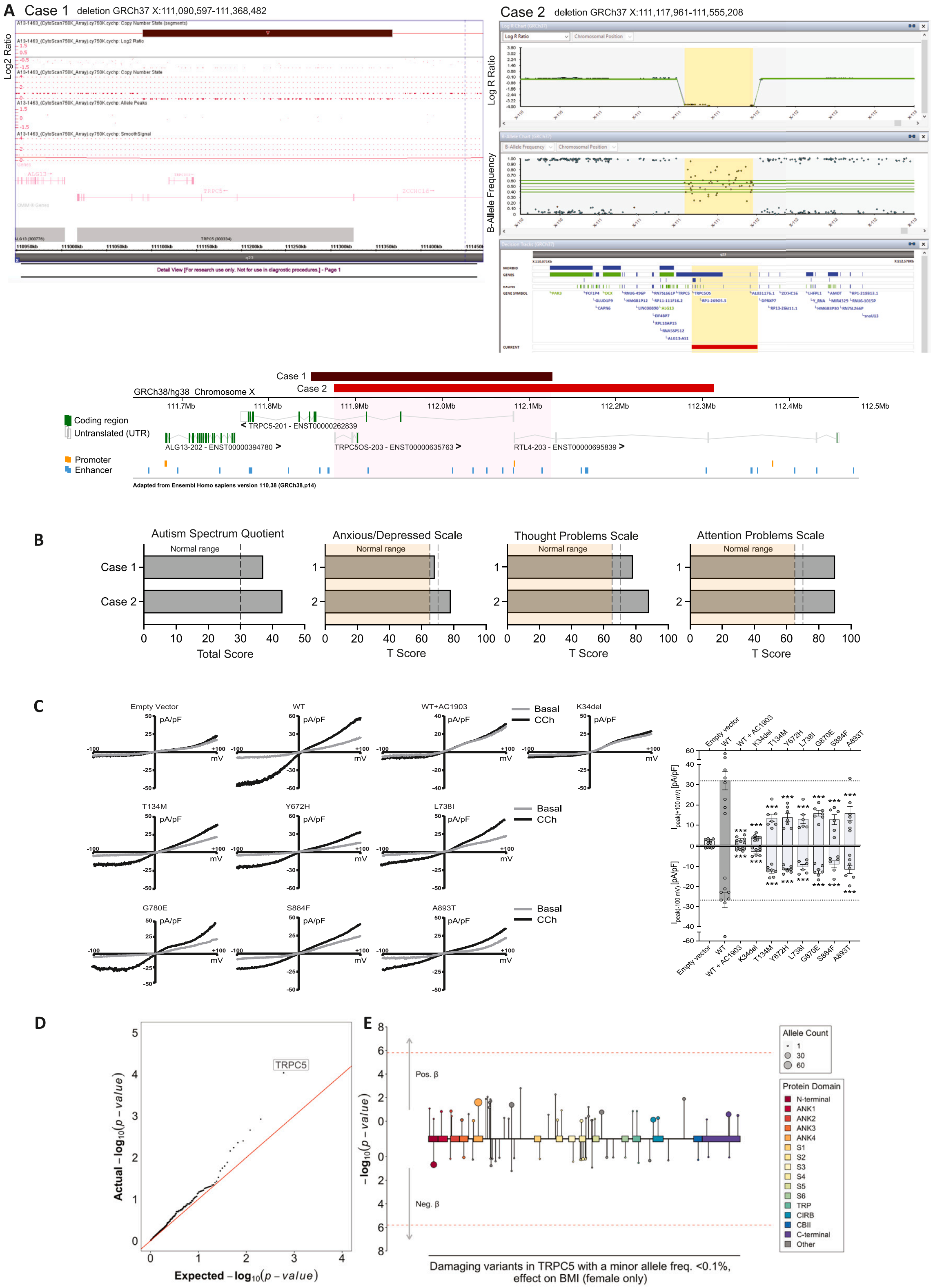

(legend on next page)

Figure S1. Genetic, molecular, and clinical features of *TRPC5* variant carriers, related to Figure 1

(A) Deletions in Cases 1 and 2 showing loss of probes and probe-level $\log_2$ ratio values obtained from array comparative genomic hybridization performed in clinical genetics laboratories. Genes and Ensembl consensus regulatory elements (cCRE) are displayed (Ensembl 110).

(B) Behavioral testing of Cases 1 and 2. Autism spectrum quotient questionnaire scores; score > 30 is consistent with autism spectrum disorder in adolescents. Child behavior checklist (CBCL) scores on the anxious/depressed, thought problems, and attention problems scales. Normal range for T scores shown (orange); scores between dashed lines are borderline clinically significant; scores above the upper dashed line are clinically significant.

(C) Functional characterization of rare *TRPC5* variants identified in the GOOS cohort showing TRPC5-mediated currents in cells transfected with empty vector, WT/mutant *TRPC5* stimulated with the acetylcholine receptor agonist, carbachol (CCh); representative current-voltage relationships shown. Data presented as mean ± SEM, *p* values determined by unpaired t test with Welch's correction; $^{***}p < 0.001$.

(D and E) Data on UK Biobank. (D) Observed associations for all genes across the X chromosome with BMI of female carriers of predicted damaging variants (black), against the expected *p* value distribution quantiles (red). (E) Variant-level associations between predicted damaging variants within *TRPC5* and BMI, in the women-only analysis. *TRPC5* variants with a minor allele frequency (MAF) less than 0.1% and annotated to either be high-confidence protein truncating variants or missense variants with a high CADD score ($\geq$25) were included. Each variant (exon in gray) is presented as an individual line extending to its association *p* value ($-\log_{10}$), indicating the direction of effect on BMI (β) in carriers of the alternate allele. Point size indicates the number of carriers of each variant (allele count) as indicated. Variants have been annotated with the protein domains in which they are located (N-terminal domain; ANK, ankyrin domains 1–4; S1–S6, transmembrane helical domains; TRP, transient receptor potential domain; CIRB, calmodulin/inositol 1,4,5-triphosphate receptor binding domain; C-terminal domain).

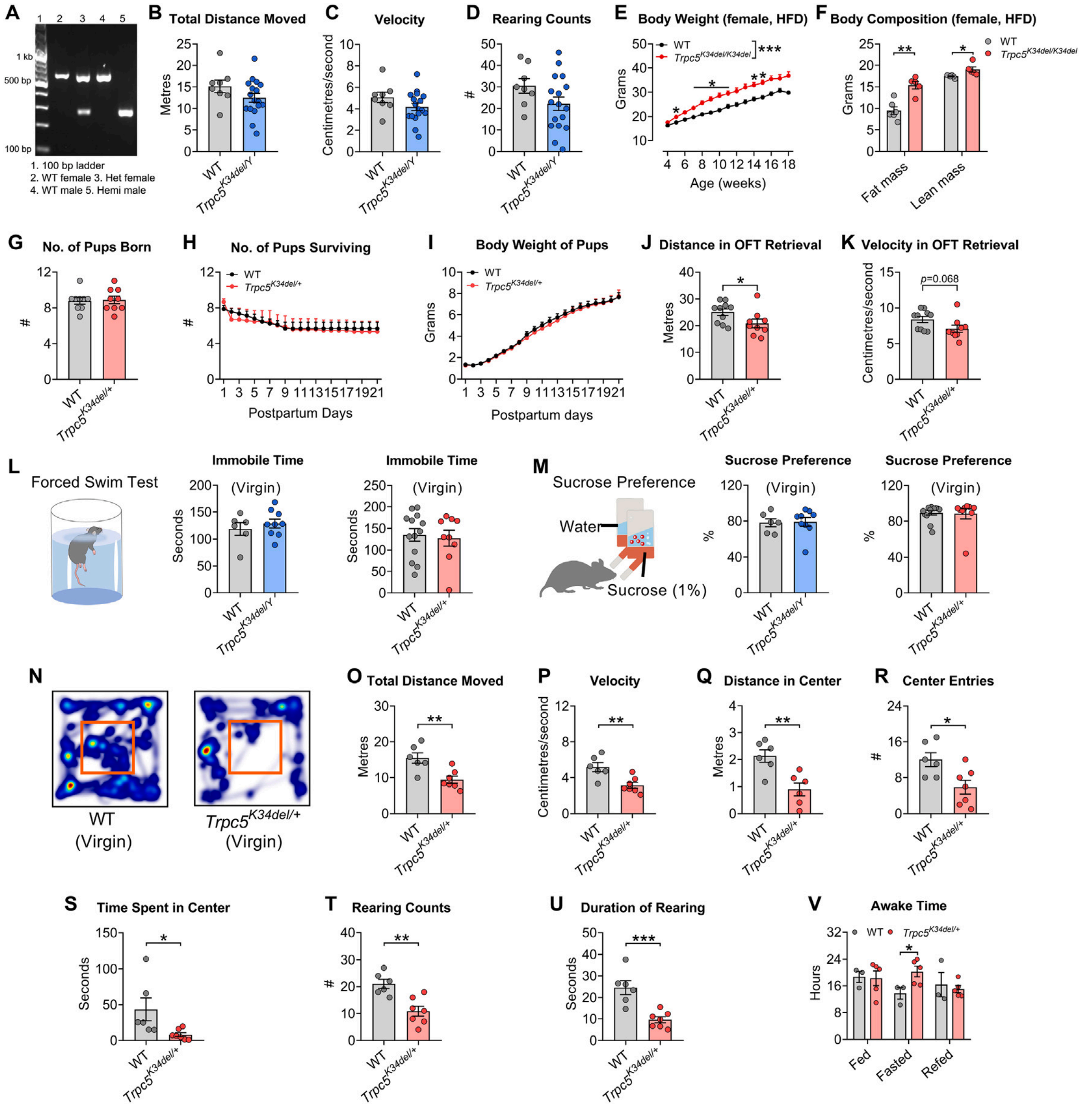

Figure S2. Metabolic and behavioral phenotype of male $Trpc5^{K34del/Y}$ and female $Trpc5^{K34del/+}$ mice, related to Figures 2 and 3

(A–F) (A) Representative genotyping results of female and male WT and $Trpc5^{K34del}$ mice. bp: base pairs. kb: 1,000 base pairs; het, heterozygous; hemi, hemizygous. Male WT and $Trpc5^{K34del/Y}$ hemizygous mice were studied in the open field arena test: total distance traveled (B), average velocity (C), total number (#) of rearing episodes (D) (n = 8–17 per group, 16 weeks of age). Body weight (E) and body composition (F) of female WT and $Trpc5^{K34del/K34del}$ homozygous mice on high-fat diet (HFD) (n = 5–8 per group).

(G–K) (G) Number (#) of pups born to WT and $Trpc5^{K34del/+}$ dams (n = 9 per group, 14 weeks of age), number of surviving pups (H), and average pup weight from postpartum days 1 to 21 (I) (n = 7–9 per group). Distance traveled for retrieval in the open field test (OFT) (J) and velocity of retrieval (K) by WT and $Trpc5^{K34del/+}$ dams (n = 9–10 per group, 14 weeks of age).

(L and M) (L) Forced swim test, immobile time in forced swim test, and (M) sucrose preference test and sucrose preference for virgin male and female WT mice compared with $Trpc5^{K34del/Y}$ and $Trpc5^{K34del/+}$ mice, respectively (males, n = 6–9 per group, 28 weeks of age; females n = 9–13 per group, 8 weeks of age).

(N–U) Virgin female WT and $Trpc5^{K34del/+}$ mice were studied in the open field arena: heatmap of movement (N), total distance traveled (O), average velocity (P), total distance traveled in the center (Q), center entries (R), time spent in center area (S), total number (#) of rearing episodes (T), and duration of rearing (U) (n = 6–7 per group, 24 weeks of age).

(V) Awake time during 24 h in the fed, fasted, and refed condition in female WT and $Trpc5^{K34del/+}$ mice (n = 3–5 per group, 24 weeks of age). Data presented as mean ± SEM, p value determined using 2-way ANOVA (E, H, and I) or unpaired t tests (B–D, F, G, and J–V). *$p < 0.05$, **$p < 0.01$, and ***$p < 0.001$. Overall difference between groups is indicated in the panel legend as appropriate.

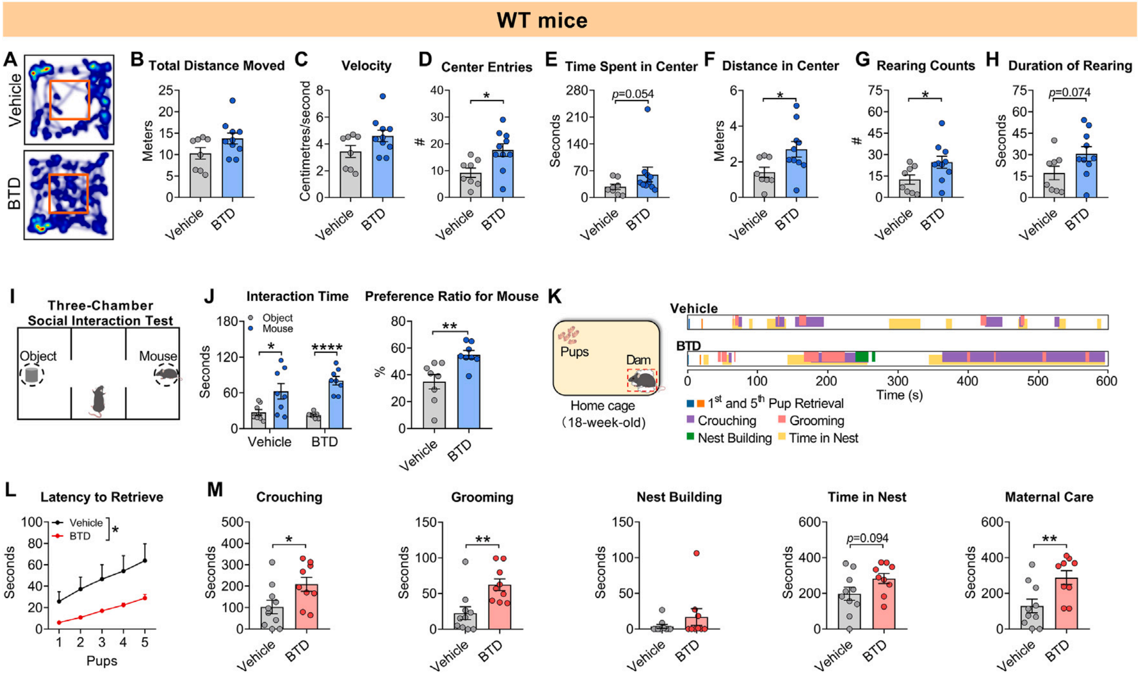

Figure S3. Effects of BTD on behavior of male and female WT mice, related to Figure 4

(A–H) WT male mice receiving vehicle or BTD injection were studied in the open field arena: heatmap of movement (A), total distance traveled (B), average velocity (C), number (#) of center entries (D), time spent in center (E), total distance traveled in the center (F), total number of rearing episodes (G), and duration of rearing (H) (*n* = 8–10 per group, 24 weeks of age).

(I) Three-chamber social interaction test used to study WT male mice receiving vehicle or BTD injection.

(J) Interaction time with object and mouse in chamber and preference ratio (mouse vs. object; *n* = 8 per group, 25 weeks of age).

(K) Maternal behavior assay in home cage and sample behavior raster plot of WT female dams receiving vehicle or BTD injection.

(L and M) (L) Latency to retrieve pups and (M) duration of crouching above pups, pup grooming and nest-building behavior, total time spent in nest, and duration of maternal care (*n* = 9–10 per group, 18 weeks of age). Data presented as mean ± SEM, *p* value determined using 2-way ANOVA (L), unpaired t tests (B–H and J), or Mann-Whitney test (M). *$p < 0.05$, **$p < 0.01$, and ****$p < 0.0001$. Overall difference between groups is indicated in the panel legend as appropriate.

CellPress
OPEN ACCESS

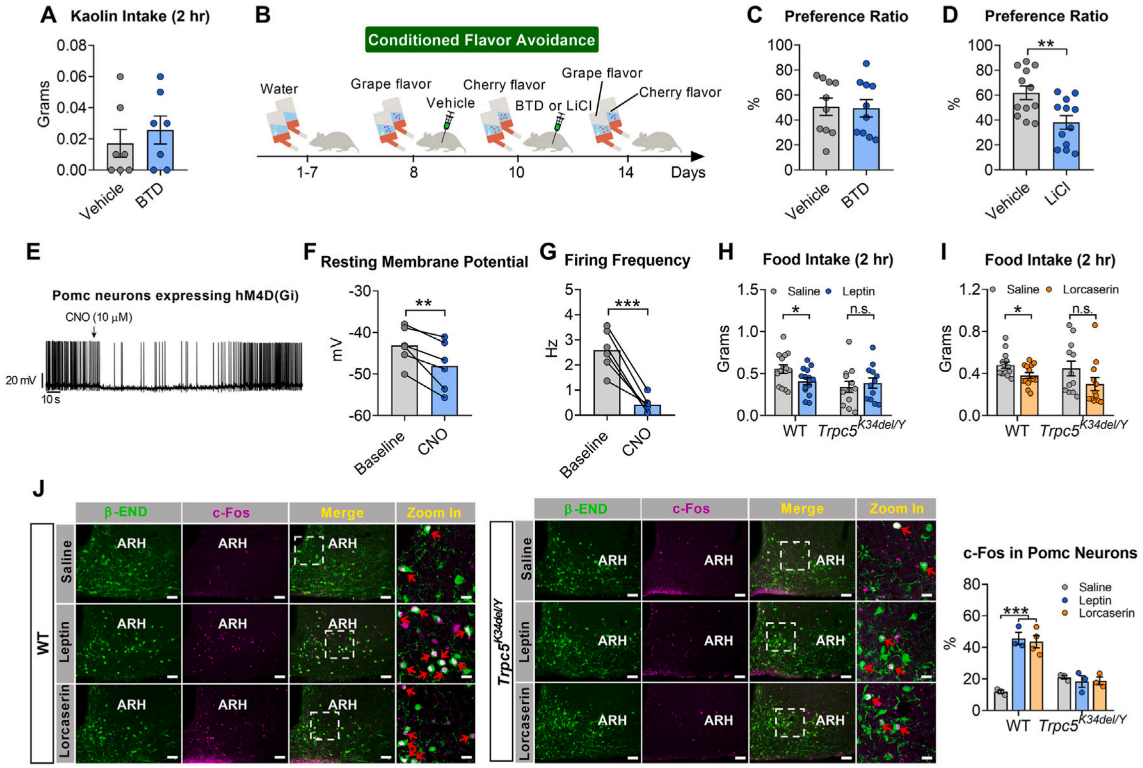

Figure S4. Trpc5-mediated activation of Pomc neurons regulates food intake, related to Figure 4

(A) Kaolin consumption within 2 h after vehicle or BTD injection at onset of the dark phase in WT male mice (n = 7 per group, 12 weeks of age).

(B–I) (B) Experimental design used for conditioned flavor avoidance (STAR Methods); BTD compared with lithium chloride (LiCl). Preference for the paired solution in vehicle and BTD-injected (C) or LiCl-injected (D) WT male mice (n = 10–12 per group, 12–16 weeks of age). Representative electrophysiological response (E), resting membrane potential (F), and firing frequency (G) in response to CNO administration on Pomc neurons expressing hM4D(Gi). Chow food intake within 2 h after leptin (H) and lorcaserin injection (I) compared with saline in male WT and $Trpc5^{K34del/Y}$ mice (n = 12–14 per group, 16 weeks of age).

(J) Representative microscopy images showing immunoreactivity of β-END (green), c-Fos (magenta), and merged images (yellow) after injection of saline, leptin, and lorcaserin in male WT (left) and $Trpc5^{K34del/Y}$ mice (right) with quantification of % of Pomc neurons (labeled by β-END) expressing c-Fos (n = 3–4 per group; 19 weeks of age). Scale bars, 25 μm. Data presented as mean ± SEM, p values determined using one-way ANOVA (J), unpaired t tests (A, C, D, H, and I), or paired t tests (F and G). *$p < 0.05$, **$p < 0.01$, and ***$p < 0.001$.

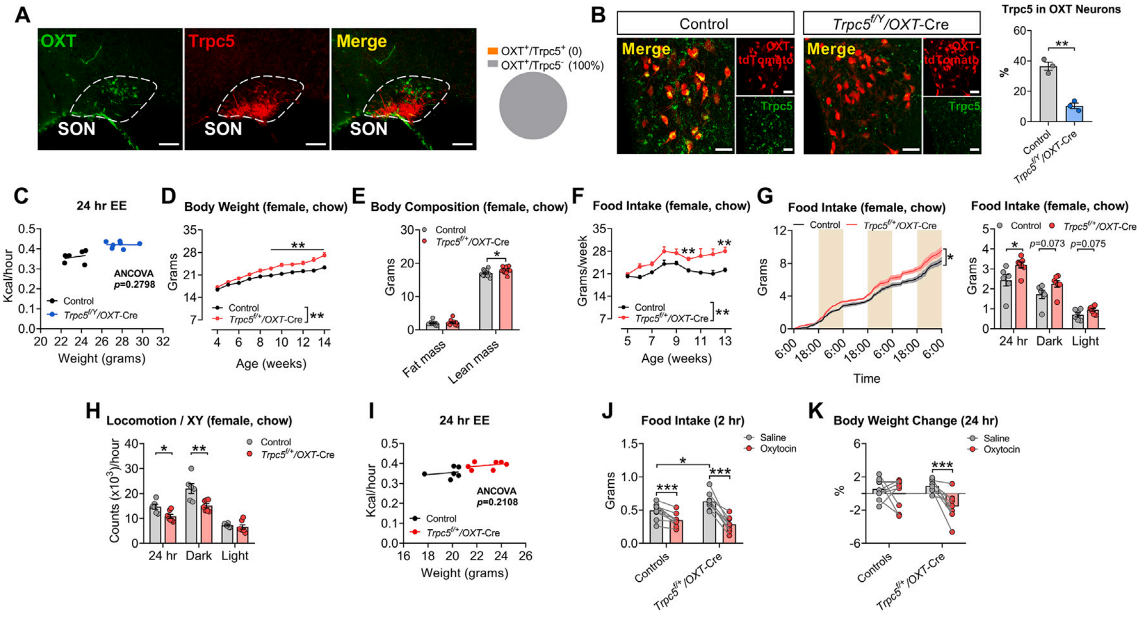

Figure S5. OXT neurons mediate effects of Trpc5 on energy homeostasis, related to Figure 5

(A) Representative microscopy images showing Trpc5 (red) and OXT (green) expression in the SON in male WT mice and quantification of Trpc5 expression within SON OXT neurons (n = 3 mice, 16 weeks of age). Scale bars, 50 μm.

(B) Representative microscopy images showing colocalization of Trpc5 (green) and OXT-tdTomato neurons (red) in the PVH and percentage of PVH OXT neurons expressing Trpc5 (n = 3 mice per group, 20 weeks of age). Scale bars, 25 μm.

(C) Regression of energy expenditure (EE) with body mass in male control and *Trpc5*$^{f/Y}$/*OXT*-Cre mice over 24 h (n = 6–7 per group, 10 weeks of age).

(D–K) Experiments in female control and *Trpc5*$^{f/+}$/*OXT*-Cre mice on chow diet (n = 7–13 per group). Body weight (D), body composition (E), weekly food intake (F), cumulative food intake during a 3-day period and food intake during 24 h, dark and light cycles (G), locomotor activity (xy axis) during 24 h, dark and light cycles, and regression of energy expenditure (EE) with body mass (n = 6–7 per group, 10 weeks of age) (H and I). Food intake (J) and body weight change (K) after saline or OXT injection in female control and *Trpc5*$^{f/+}$/*OXT*-Cre mice (n = 9 per group, 20 weeks of age). Data presented as mean ± SEM, p values determined using 2-way ANOVA (D, F, and G), unpaired t tests (B, E, G, H, J, and K), or paired t tests (J and K). *$p < 0.05$, **$p < 0.01$, and ***$p < 0.001$. Overall difference between groups is indicated in the panel legend as appropriate.

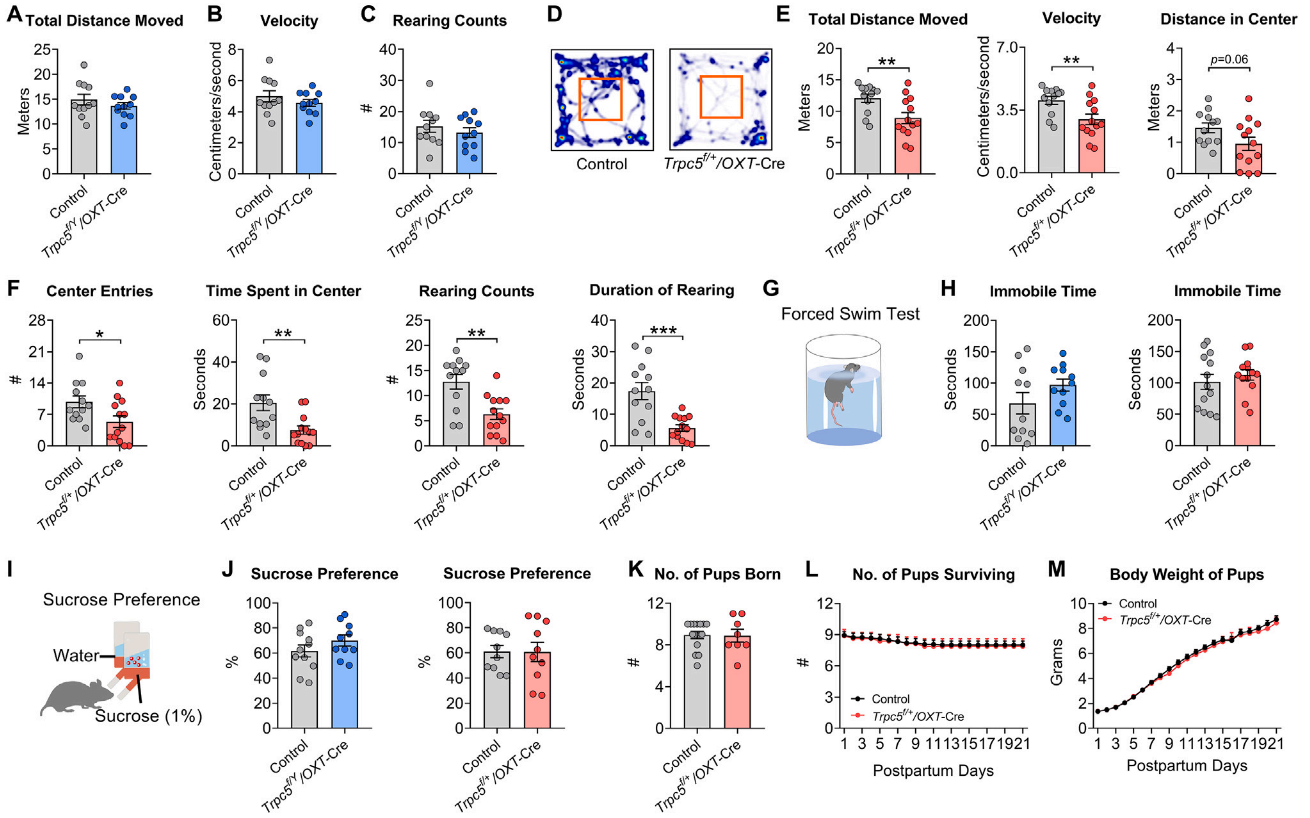

Figure S6. OXT neurons mediate effects of Trpc5 on behavior, related to Figure 6

(A–C) Virgin male control and *Trpc5*$^{f/Y}$/*OXT*-Cre mice were studied in the open field test: total distance traveled (A), average velocity (B), and total number (#) of rearing episodes (C) (*n* = 11 per group, 12 weeks of age).

(D–F) Virgin female control and *Trpc5*$^{f/+}$/*OXT*-Cre mice were studied in the open field test: heatmap of movement (D), total distance traveled, average velocity, total distance traveled in the center (E), center entries, time spent in center, total number (#) of rearing episodes, and duration of rearing (F) (*n* = 12–13 per group, 12 weeks of age).

(G) Forced swim test.

(H) Immobile time in forced swim test for (left) virgin male control and *Trpc5*$^{f/Y}$/*OXT*-Cre mice (*n* = 11 mice per group, 12 weeks of age) and (right) virgin female control and *Trpc5*$^{f/+}$/*OXT*-Cre mice (*n* = 13 mice per group, 13 weeks of age).

(I) Sucrose preference test.

(J) Sucrose preference for virgin male control and *Trpc5*$^{f/Y}$/*OXT*-Cre mice (left) (*n* = 10–11 mice per group, 12 weeks of age) and virgin female control and *Trpc5*$^{f/+}$/*OXT*-Cre mice (right) (*n* = 10 mice per group, 13 weeks of age).

(K–M) (K) Number of pups born to control and *Trpc5*$^{f/+}$/*OXT*-Cre dams, (L) number of surviving pups, and (M) average pup weight from postpartum days 1 to 21 (*n* = 8–15 per group, 17 weeks of age). Data presented as mean ± SEM, *p* values determined using unpaired t tests (A–C, E, F, H, J, and K) or using 2-way ANOVA (L and M). *$p < 0.05$, **$p < 0.01$, and ***$p < 0.001$.

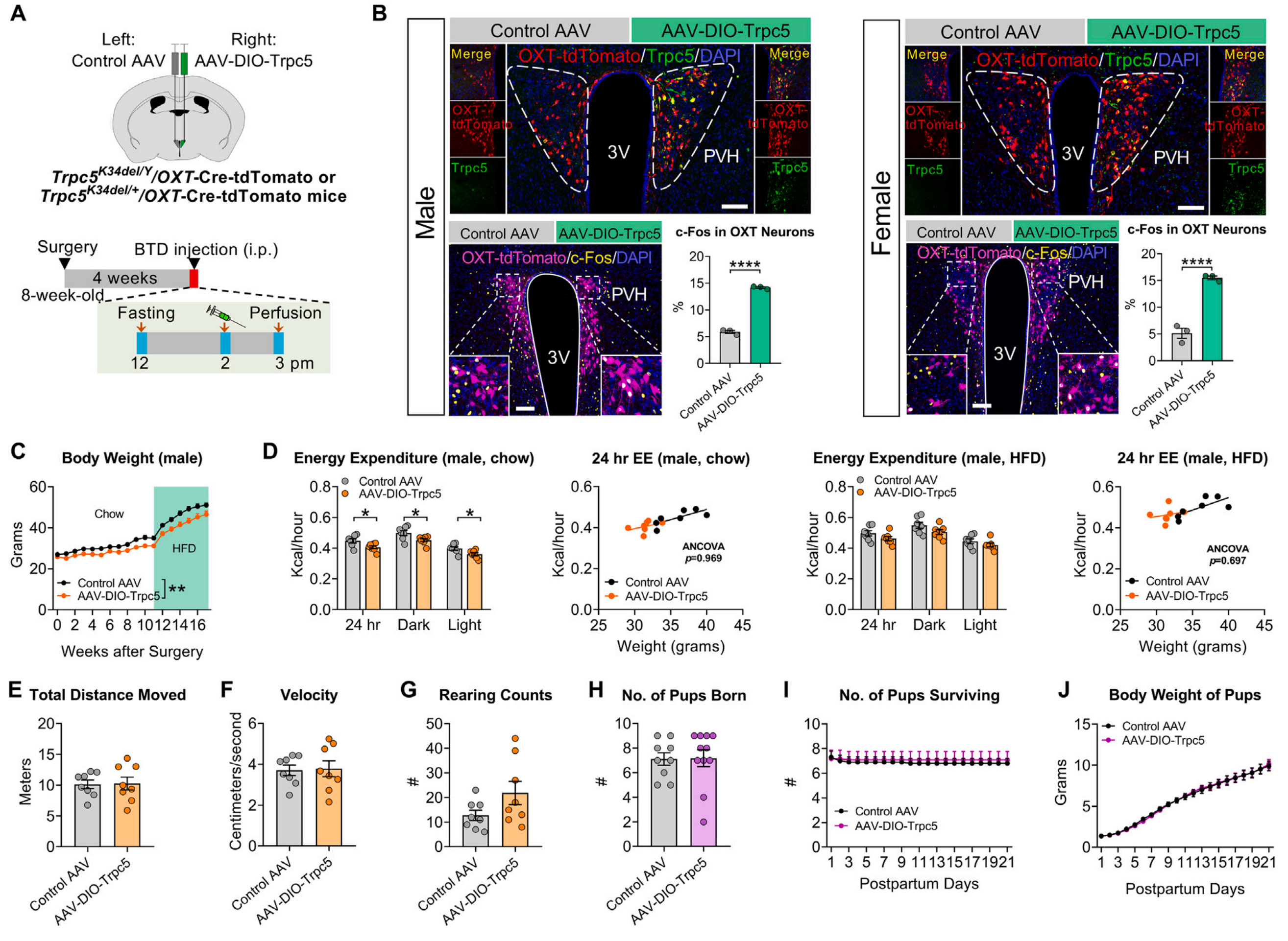

Figure S7. Restoration of Trpc5 in PVH OXT neurons improves metabolic and behavioral phenotypes due to Trpc5 deficiency, related to Figure 7

(A) Experimental paradigm to validate the AAV-DIO-Trpc5 and analyze restoration of Trpc5 expression in OXT neurons in response to BTD administration.

(B) Expression of Trpc5 and c-Fos in OXT neurons after BTD injection in male *Trpc5*$^{K34del/Y}$/*OXT*-Cre/Rosa26-LSL-tdTomato (left) and female *Trpc5*$^{K34del/+}$/*OXT*-Cre/Rosa26-LSL-tdTomato mice (right) receiving control AAV in the left and AAV-DIO-Trpc5 in the right PVH (*n* = 3 mice, 12 weeks of age). Scale bars, 50 µm; 3V, third ventricle; DAPI, nuclear stain.

(C) Body weight of male *Trpc5*$^{K34del/Y}$/*OXT*-Cre mice receiving control AAV or AAV-DIO-Trpc5 during chow and HFD feeding (*n* = 8 per group).

(D) Energy expenditure (EE) during 24 h, dark and light cycles, and regression of EE with body mass on chow and HFD (*n* = 7 per group, 22 weeks of age).

(E–G) Male *Trpc5*$^{K34del/Y}$/*OXT*-Cre mice receiving control AAV or AAV-DIO-Trpc5 were studied in the open field test: total distance traveled (E), average velocity (F), total number (#) of rearing episodes (G) (*n* = 8 per group, 13 weeks of age).

(H–J) (H) Number of pups born to *Trpc5*$^{K34del/+}$/*OXT*-Cre mice receiving control AAV or AAV-DIO-Trpc5 (*n* = 9–11 per group, 24 weeks of age), number of surviving pups (I), and average pup weight from postpartum days 1 to 21 (J). Data presented as mean ± SEM, *p* values determined using 2-way ANOVA (C, I, and J), unpaired t tests (B, D, and E–H). *$p < 0.05$, **$p < 0.01$, ****$p < 0.0001$. Overall difference between groups is indicated in the panel legend as appropriate.
